# The Impact of Health Literacy–Sensitive Design and Heart Age in a Cardiovascular Disease Prevention Decision Aid: Randomized Controlled Trial and End-User Testing

**DOI:** 10.2196/34142

**Published:** 2022-04-15

**Authors:** Carissa Bonner, Carys Batcup, Julie Ayre, Erin Cvejic, Lyndal Trevena, Kirsten McCaffery, Jenny Doust

**Affiliations:** 1 School of Public Health Faculty of Medicine and Health University of Sydney Sydney Australia; 2 School of Public Health Faculty of Medicine University of Queensland Brisbane Australia

**Keywords:** decision aids, shared decision-making, risk communication, heart age, cardiovascular disease prevention, behavior change, health literacy

## Abstract

**Background:**

Shared decision-making is an essential principle for the prevention of cardiovascular disease (CVD), where asymptomatic people consider lifelong medication and lifestyle changes.

**Objective:**

This study aims to develop and evaluate the first literacy-sensitive CVD prevention decision aid (DA) developed for people with low health literacy, and investigate the impact of literacy-sensitive design and heart age.

**Methods:**

We developed a standard DA based on international standards. The standard DA was based on our existing general practitioner DA. The literacy-sensitive DA included simple language, supporting images, white space, and a lifestyle action plan. The control DA used Heart Foundation materials. A randomized trial included 859 people aged 45-74 years using a 3 (DA: standard, literacy-sensitive, control) ×2 (heart age: heart age + percentage risk, percentage risk only) factorial design, with outcomes including prevention intentions and behaviors, gist and verbatim knowledge of risk, credibility, emotional response, and decisional conflict. We iteratively improved the literacy-sensitive version based on end-user testing interviews with 20 people with varying health literacy levels.

**Results:**

Immediately after the intervention (n=859), there were no differences in any outcome among the DA groups. The heart age group was less likely to have a positive emotional response, perceived the message as less credible, and had higher gist and verbatim knowledge of heart age risk but not percentage risk. After 4 weeks (n=596), the DA group had better gist knowledge of percentage risk than the control group. The literacy-sensitive DA group had higher fruit consumption, and the standard DA group had better verbatim knowledge of percentage risk. Verbatim knowledge was higher for heart age than for percentage risk among those who received both.

**Conclusions:**

The literacy-sensitive DA resulted in increased knowledge of CVD risk and increased fruit consumption in participants with varying health literacy levels and CVD risk results. Adding heart age did not increase lifestyle change intentions or behavior but did affect psychological outcomes, consistent with previous findings. This tool will be integrated with additional resources to improve other lifestyle outcomes.

**Trial Registration:**

Australian New Zealand Clinical Trials Registry ACTRN12620000806965; https://tinyurl.com/226yhk8a

## Introduction

### Background

Prevention of cardiovascular disease (CVD) includes lifestyle interventions and medication for those at highest risk who are most likely to benefit. An absolute risk approach is supported by clinical evidence and endorsed by many national guidelines worldwide [1-5]. The absolute risk of a heart attack or stroke in the next 5-10 years can be assessed using widely available calculators [1]; however, these tools are substantially underused in practice [6-11]. Providing medication to high-risk and not low-risk patients is a cost-effective approach [6]. However, up to 75% of high-risk patients do not receive recommended medication to prevent death and disability from CVD, whereas 25% of low-risk patients take medication they are very unlikely to benefit from [7]. Recent guideline changes have led to calls for a shared decision-making approach to ensure that medication prescription for blood pressure and cholesterol is more in line with patient values [12-14].

Health literacy also plays a role in CVD prevention. Low health literacy is common in many countries, with estimates ranging from 36% to 60% of the population in Australia, Europe, and the United States [15-17]. This is associated with poorer self-management, less access to the health system, increased incidence of chronic diseases, including CVD, and increased mortality [18]. Therefore, it is important to engage this group in communication strategies for CVD prevention. This requires changes to the design of web-based patient resources, as many Australians seek health information on the web [19,20], but fewer than 1% of health information websites meet the recommended readability levels. Grade 8 is recommended to meet the needs of people with varying health literacy [21,22].

Some countries have used web-based CVD risk assessment tools for absolute risk and heart age to engage consumers in CVD prevention, with millions of users worldwide [23-26]. However, our systematic review of 73 web-based CVD risk assessment tools available to consumers found that they were not suitable for people with lower health literacy: their readability level was too high; they frequently used unexplained medical terms; few used best practice risk communication formats such as frequencies in icon arrays; and they rated poorly on actionability (ie, clarity in instructions of what actions or steps to take), which makes it difficult for the average person to know what to do about the risk assessment result [27]. Our review of 25 web-based decision aids (DAs) for CVD prevention found similar issues with understandability and actionability [28], and few included lifestyle changes as an option to reduce risk, with many focusing on medication only.

There are several evidence-based strategies to address the issue of communicating CVD risk to people with lower health literacy, such as:

1. Use literacy-sensitive design to improve the readability of health information and reduce the cognitive load of action plans for behavior change [29-31].
2. Use best practice risk communication formats to explain abstract probabilities (eg, 16%) using icon arrays and more concrete frequencies (eg, 16 out of 100 people like you) [32-35].
3. Use patient DAs to improve understanding and decision-making, including both lifestyle change and medication, as clear actions that patients can take to reduce their CVD risk [29,36,37].

### Objectives

This study aims to develop and test a new consumer engagement tool for CVD prevention based on the aforementioned strategies to address the needs of Australians with different levels of health literacy. It builds on our previous development of a general practitioner (GP)-focused risk calculator and DA [38] and evaluation of the national heart age calculator [26].

## Methods

### Ethics Approval

This study received ethics approval from the Human Research Ethics Committee of the University of Sydney (project number 2019/774).

### Stage 1: Develop Consumer Engagement Tool

In stage one, we developed a literacy-sensitive version of our existing GP DA [39], which calculates 5-year risk of a CVD event based on current guidelines [1] and shows the effects of 9 lifestyle, medication, and supplement interventions [38]. This was based on previous reviews and evaluations of 73 CVD risk calculators and 25 CVD prevention DAs, which identified tools for many different CVD models, but none that matched Australian guidelines and best practice communication principles [27,28]. We added heart age to the Australian absolute CVD risk calculation based on published methods from New Zealand, both of which use the 5-year Framingham equation [40]. The literacy-sensitive design included simple language, supporting images, and white space to improve readability and understandability [30]. The text within this DA was evaluated using the Sydney Health Literacy Editor, a tool that automatically applies readability and actionability criteria to the text [41]. On the basis of this feedback, the final tool met the recommended grade 8 level. The literacy-sensitive version also included a novel action plan format developed by our team, which has been shown to reduce unhealthy lifestyle behaviors among people with low health literacy [31]. We added options for physical activity and smoking to the existing tools to reduce unhealthy snacking, drawing on previous literature on effective if-then plans in these areas. If-then plans help people identify an important environment context or trigger in which they find that they often carry out an unwanted behavior and to identify a new behavior that can be substituted for the unwanted behavior. These 2 components are formulated into an *if-then* statement or plan; for example, *If I find myself eating unhealthy snacks when drinking a cup of tea, then I will eat a piece of fruit instead.* In this study, we used an if-then format called a *volitional help sheet*, which prompts the person with predefined *if* and *then* statements [42-44].

### Stage 2: Randomized Trial to Identify Best Formats for Low Versus High Health Literacy Design

#### Overview

**Figure 1:**
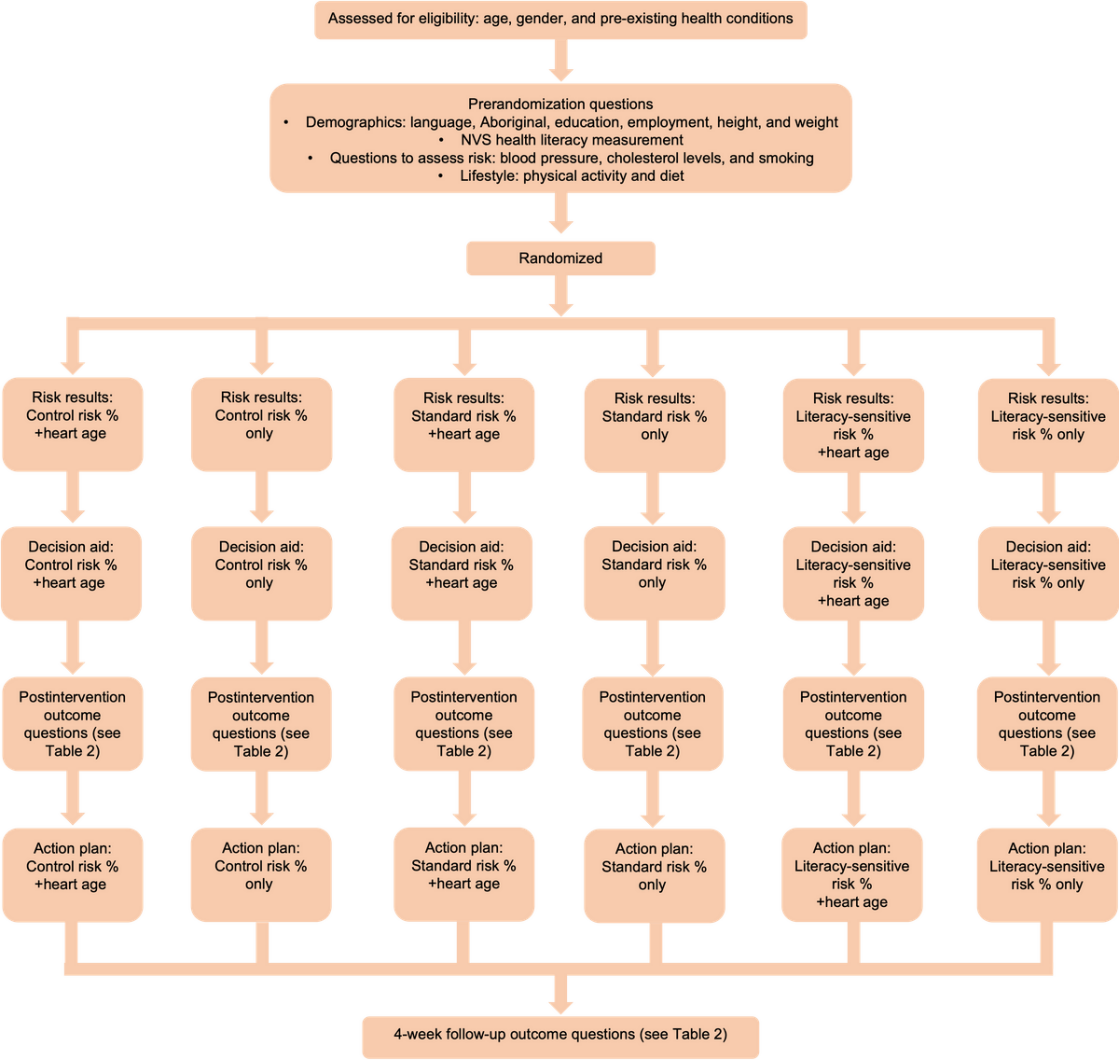
Study design. NVS: Newest Vital Signs.

**Figure 2:**
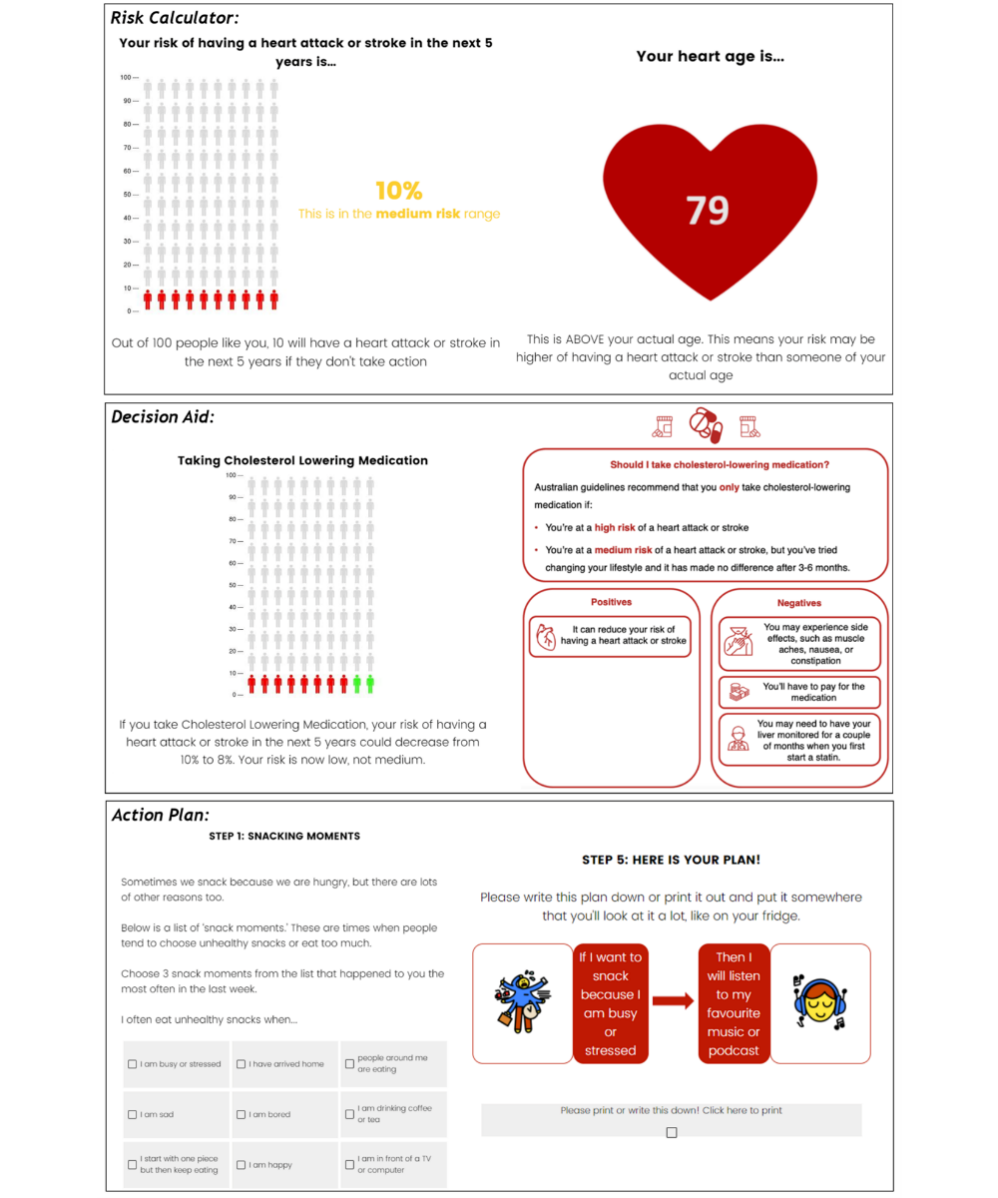
Example risk calculator, decision aid, and action plan (literacy-sensitive heart age version).

The randomized trial was based on a 3×2 factorial, between-subject design to test the effect of literacy-sensitive design (literacy-sensitive DA, standard DA, or control: Heart Foundation patient information) and risk format (explaining CVD risk only [as a percentage risk], or CVD risk percentage+heart age) on psychological and behavioral outcomes. See Table 1 and Figure 1 for study design and Figure 2 and Multimedia Appendix 1 for example intervention content. The trial was preregistered at the Australian New Zealand Clinical Trials Registry (ACTRN12620000806965).

**Table 1:** The 2×3 study design.

Group	Risk results	Decision aid (DA)	Action plan
Control HF^a^ information—risk percentage (+heart age)	Absolute percentage risk shown in the design of HF risk calculator results [45]. For participants in the heart age group, heart age also shown in the design of HF heart age calculator.	In the design of the National Vascular Disease Prevention Alliance risk calculator [45], participants can change any risk factors and are then presented with their risk percentage compared with their *updated risk* based on the changes they made to the risk factors. They are then advised to book in for a heart health check with their doctor.	Participants receive feedback on their blood pressure, cholesterol, and BMI. Then they are prompted to select a topic to see more information about (diet, exercise or smoking). This information is taken from the HF website [46-48].
Standard DA—risk percentage (+heart age)	Absolute percentage risk shown alongside an icon array, with the number of icons in red (out of 100 gray icons), demonstrating the risk percentage. For participants in the heart age group, heart age also shown in the design of HF heart age calculator.	Participants were asked to choose an option to reduce their risk, out of nine potential options in three categories (medication, lifestyle changes, and supplements). Once they chose an option, they were shown an icon array with the new risk in red and the difference between their current and new risk in green. They were then shown information from our current CVD^b^ risk website about the option they chose as well as a table of the benefits and harms of that choice [39].	Participants had to choose a lifestyle behavior change to make (smoking, exercise, or diet) and then create a goal. They were then guided through creating a *SMART*^c^ *goal* design plan, taken from our current CVD risk website [39].
Literacy-sensitive DA—risk percentage (+heart age)	Absolute percentage risk shown alongside an icon array, with the number of icons in red (out of 100 gray icons), demonstrating the risk percentage. For participants in the heart age group, heart age also shown with more explanation than control and standard DA conditions.	The same as for the standard DA; however, the information and benefits and harms were edited to be appropriate for all levels of health literacy; for example, by introducing white space, images, and reducing the readability level.	Participants were prompted to change their smoking, exercise, or snacking habits. They were then guided through creating an action plan based on implementation intentions or *if-then* plans. The snacking action plan was previously developed by our team [31], and the exercise and smoking plans were in the same design using research in those areas [42,43].

^a^HF: Heart Foundation.

^b^CVD: cardiovascular disease.

^c^SMART: Specific, Measurable, Achievable, Realistic, and Timely.

#### Recruitment

A national sample was recruited through Qualtrics (Qualtrics Inc), a web-based social research agency, with stratified sampling based on gender and age groups (5-year age groups from 45 to 74 years). Participants completed a CVD risk assessment based on the Australian guidelines and New Zealand approach to calculate heart age [1,40]. If blood pressure or cholesterol were not known, the average by age and gender based on non-diabetic participants in the AusDiab cohort was used (accessed via author JD), and all participants were advised to see a GP for a more accurate risk assessment. Participants with established CVD or those taking CVD prevention medications were excluded. Duplicate IP addresses were replaced, and stratified sampling was relaxed with additional quality checks added if hard-to-reach groups did not reach the quota after 2 weeks.

#### Measures

Established measures were used for the primary outcome of behavioral intentions (validated theory of planned behavior scale applied to smoking, diet, exercise, and GP visit) [49-51]. Secondary outcomes included self-reported behavior after 4 weeks compared with national guidelines for diet and physical activity [50,51], gist and verbatim knowledge (absolute risk percentage and heart age), emotional response using a validated scale (3 positive emotions, eg, hopeful, and 3 negative emotions, eg, anxious) [52], credibility of the information (that the information is personally relevant) [53], and decision conflict scale (uncertainty in decision-making) [54]. Details are presented in Table 2.

**Table 2:** Psychological and behavioral outcomes measured in the analyses.

Outcome and items		Response scale	Immediately after the intervention	4-week follow-up
**Lifestyle intentions** **[49]**				
	I intend to smoke less/improve my diet/increase the amount of physical activity I do in the next 4 weeks (average 2-3 items depending on smoking)	1=strongly disagree to 7=strongly agree	✓^a^	
**Medication intentions** **[49]**				
	I intend to talk to my GP^b^ about taking blood pressure lowering medication/cholesterol lowering medication/aspirin in the next 4 weeks (average 3 items)	1=strongly disagree to 7=strongly agree	✓	
**Supplement intentions** **[49]**				
	I intend to take fish oil/multivitamin/antioxidant supplements in the next 4 weeks (average 3 items)	1=strongly disagree to 7=strongly agree	✓	
**Credibility** **[53]** **(Cronbach α=.89)**				
	I felt that the numbers received were “my numbers”;	1=strongly disagree to 7=strongly agree	✓	
	I found the results to be written personally for me;			
	I felt that the information was relevant to me;			
	I felt that the information was designed specifically for me			
**Emotion (positive Cronbach α=.81; negative Cronbach α=.85)** **[52]**				
	My results made me feel: Positive subscale: hopeful/optimistic/enthusiastic; Negative subscale: afraid/anxious/worried	0=none of this feeling to 10=a lot of this feeling	✓	.
**Gist knowledge of percentage risk**				
	My risk level for having a heart attack or stroke in the next 5 years was	Low/medium/high/I don't know	✓	✓
**Verbatim knowledge of percentage risk**				
	My percentage risk of having a heart attack or stroke in the next 5 years was	Numerical/I don't know	✓	✓
**Gist knowledge of heart age**				
	My heart age result was	Below my actual age/the same as my actual age/above my actual age/I wasn't shown my heart age/I don't know	✓	✓
**Verbatim knowledge of heart age**				
	My heart age was	Numerical/I don't know	✓	✓
**Decisional conflict** **[54]**				
	Do you feel sure about the best choice for you?	Yes/no	✓	
	Do you know the benefits and risks of each option?	Yes/no	✓	
	Are you clear about which benefits and risks matter most to you?	Yes/no	✓	
	Do you have enough information to make a choice?	Yes/no	✓	
**Smoking^c^**				
	Do you currently smoke cigarettes?	Yes/no		✓
	In the last week, how many cigarettes did you usually smoke per day?	Numerical (if yes)		✓
**Physical activity [50]^c^**				
	In the last week, how many times did you do 20 minutes or more of vigorous-intensity physical activity that made you sweat or puff and pant?	0-10+ (assessed as adequate/inadequate against Australian diet guidelines)		✓
	In the last week, how many times did you do 30 minutes or more of moderate-intensity physical activity or walking that increased your heart rate or makes you breathe harder than normal?	0-10+ (assessed as adequate/inadequate against Australian diet guidelines)		✓
**Diet [51]^c^**				
	In the last week, how many serves of fruit did you usually eat per day?	0-10+ (with examples of serves provided; assessed as adequate/inadequate against Australian diet guidelines)		✓
	In the last week, how many serves of vegetables did you usually eat per day?	0-10+ (with examples of serves provided; assessed as adequate/inadequate against Australian diet guidelines)		✓
	In the last week, how many serves of unhealthy snacks did you usually eat per day?	0-10+ (with examples of serves provided; assessed as adequate/inadequate against Australian diet guidelines)		✓
	In the last week, how much soft drink, cordial or sports drinks do you usually drink per day?	0-10+ (with examples of serves provided; assessed as adequate/inadequate against Australian diet guidelines)		✓
**Seeing a doctor**				
	Have you discussed your risk of heart disease with a doctor in the last 4 weeks? (including blood pressure, cholesterol or lifestyle change)	Yes/no		✓
	Have you made an appointment to discuss your risk of heart disease with a doctor? (including blood pressure, cholesterol or lifestyle change)	Yes/no		✓
**Helpline**				
	Have you used the Heart Foundation helpline for more lifestyle change support?	Yes/no		✓

^a^The tick demonstrates in which survey this outcome was measured.

^b^GP: general practitioner.

^c^Also asked before the intervention, with preintervention behavior controlled for in the analyses.

#### Analysis

*A priori* sample size calculations determined that 85 participants per randomized group (total n=510) would yield 90% power to detect a moderate effect size of Cohen *d*=0.5 (a standardized difference; this generic effect size estimate was selected because of the absence of similar trials on which to base calculations) in the primary outcome of intention to change lifestyle or any of the secondary outcomes, assuming a 2-sided Cronbach *α* of .05. We aimed to recruit an additional 20% more cases to account for potential missing values, totaling 600 participants (100 per group) at follow-up. This sample was inflated for recruitment to 850 participants to account for potential attrition of up to 30% between the intervention and follow-up.

Continuous outcome variables were modeled using linear regression. Dichotomous outcomes were analyzed using modified Poisson regression (using a log-link function with robust error variances). Ordinal logistic regression was used to analyze the ordered categorical outcomes. Count outcomes were modeled using negative binomial regression. All regression models included the DA group (literacy-sensitive DA, standard DA, or basic Heart Foundation patient information) and risk format (CVD risk percentage only or CVD risk percentage+heart age) as categorical variables and controlled for health literacy adequacy (categorical based on the Newest Vital Signs measure [55,56]: low, moderate, or adequate) and absolute risk (percentage). Postintervention and follow-up outcomes were analyzed separately, with follow-up analyses controlling for preintervention values where available. Pairwise comparisons were conducted to test these hypotheses. We also conducted exploratory analyses of potential differences in DA effects between health literacy levels by including a literacy-sensitive-by-DA interaction term and heart age category for heart age groups (younger or same vs older in stratified analyses). Chi-square test for paired proportions by McNemar was used to compare knowledge of heart age versus percentage risk among those who saw both. Analyses were conducted using Stata (version 16.1; StataCorp). No adjustments were made for multiple comparisons.

#### Hypotheses

1. The two DA formats will be more effective (ie, increase lifestyle change intentions or behavior and knowledge of risk without reducing credibility) than the standard Heart Foundation information.
2. The literacy-sensitive DA will be more effective than the standard DA for everyone (not just people with lower health literacy).
3. Adding heart age to absolute risk will be more effective than absolute risk alone.

### Stage 3: Iterative End-User Testing With Varying Health Literacy Levels

As part of the follow-up survey, participants in the trial were invited to opt-in to a *think aloud* interview to provide further end-user testing and feedback for the literacy-sensitive version of the intervention. From the 27 participants who provided email addresses, 20 (74%) participants were selected to represent a range of ages, genders, risk levels, and health literacy levels. Participants went through the risk calculator in full while saying out loud everything they were thinking; for example, any areas of confusion. Further questions were asked to prompt more discussion or elaboration. Transcripts were thematically coded and discussed after each set of 4-5 interviews, and improvements were made to the intervention before the next set of interviews. We conducted 2 rounds of interviews with people with low health literacy as our key target group (8/20, 40%) and then tested the improved tool with people who had higher health literacy to ensure that it was suitable for these users in another 2 rounds (12/20, 60%).

## Results

### Stage 1

**Figure 3:**
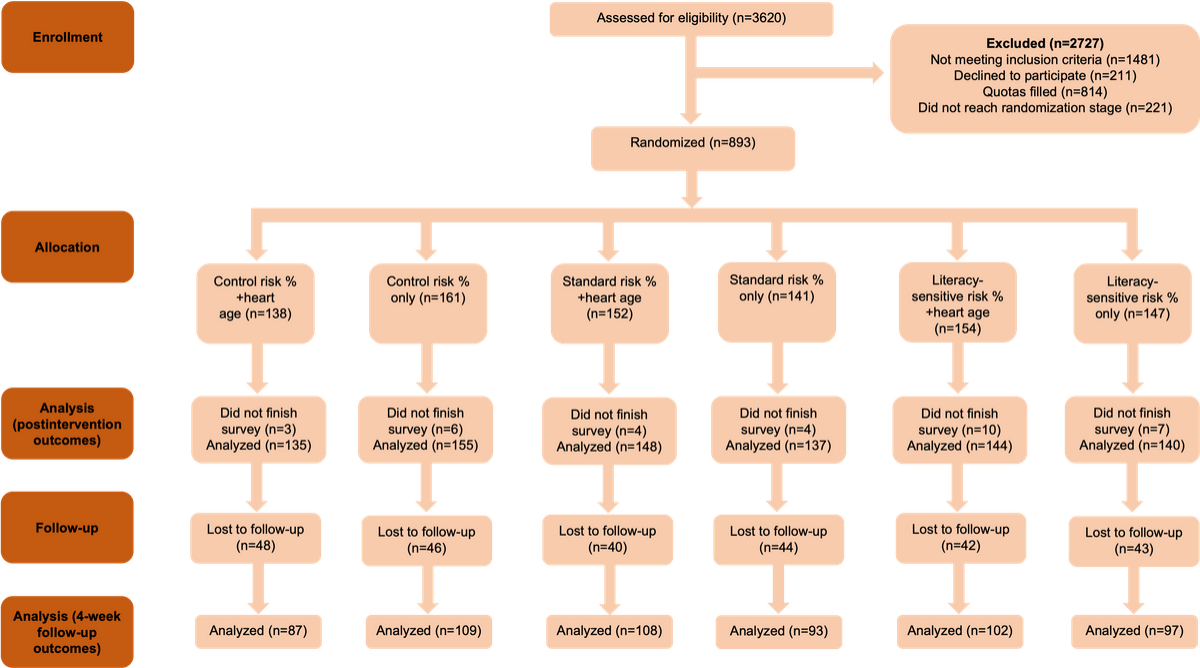
CONSORT (Consolidated Standards of Reporting Trials) diagram.

We used the question format and style of the current national heart age calculator as the basis for the risk factor questions in all groups, as well as the heart age presentation on that tool. The CVD risk results and DA were presented based on (1) our existing GP DA tool [39] (standard DA group), (2) a simplified version of the standard DA with supporting images (literacy-sensitive DA group; Figure 2), and (3) the current risk calculator from the National Vascular Disease Prevention Alliance [45]. See Multimedia Appendix 1 for example intervention content in each group.

### Stage 2

#### Overview

The CONSORT (Consolidated Standards of Reporting Trials) diagram is shown in Figure 3, and the characteristics of all the participant groups in the intervention are shown in Table 3. We conducted a soft launch with 100 participants to check that we had an adequately low health literacy sample and adequate follow-up considering the COVID-19 disruptions in 2020 before proceeding with the full trial with no changes to the preregistered method. We recruited 859 participants for the intervention (including the 100 in the soft launch), with a target of 600 at the 4-week follow-up, for which we recruited 596 participants. The characteristics were similar among the groups for age and gender but some differences were observed for health literacy (relating to education) and absolute risk (relating to smoking and heart age); therefore, these 2 factors were controlled for in the analyses. In terms of dropout, there was no difference in the randomized DA group (*P=*.71), randomized to heart age (*P=*.91), health literacy level (*P=*.69), CVD risk level (*P=*.56), or heart age result (*P=*.30) between those who returned for follow-up and those who did not. The outcomes by trial group are shown in Table 4, and the analyses for each of the 3 hypotheses are shown in Tables 5-7.

**Table 3:** Trial participant characteristics by randomized group.

Characteristics			Decision aid group			Heart age group	
			Control (n=290)	Standard (n=285)	Literacy-sensitive (n=284)	Risk percentage only (n=432)	Risk percentage+heart age (n=427)
**Demographics**							
	Age (years), mean (SD)		59.9 (8.7)	59.6 (8.2)	58.3 (8.7)	58.8 (8.6)	59.8 (8.5)
	Heart age (years), mean (SD)		60.7 (14.7)	60.9 (13.1)	58.5 (13.7)	58.9 (14.0)	61.2 (13.7)
	**Sex**						
		Male, n (%)	137 (47.2)	147 (51.6)	142 (50)	213 (49.3)	213 (49.9)
		Female, n (%)	153 (52.8)	138 (48.4)	142 (50)	219 (50.7)	214 (50.1)
	Education (university degree), n (%)		149 (51.4)	133 (46.7)	145 (51.1)	218 (50.5)	209 (48.9)
	Inadequate health literacy, n (%)		63 (21.7)	77 (27)	66 (23.2)	103 (23.8)	103 (24.1)
**Clinical characteristics**							
	Knew their cholesterol, n (%)		41 (14.1)	41 (14.4)	34 (12)	59 (13.7)	57 (13.3)
	Total cholesterol^a^ (mg/dL), mean (SD)		4.9 (1.3)	4.9 (1.5)	4.4 (1.4)	4.6 (1.3)	4.8 (1.5)
	High-density lipoprotein cholesterol^a^ (mg/dL), mean (SD)		2.6 (1.3)	2.6 (1.2)	2.8 (1.3)	2.8 (1.3)	2.6 (1.2)
	Knew their blood pressure, n (%)		102 (35.2)	106 (37.2)	98 (34.5)	162 (37.5)	144 (33.7)
	Systolic blood pressure^a^ (mm Hg), mean (SD)		123.9 (15.1)	127.0 (14.8)	124.9 (14.8)	123.9 (14.7)	126.8 (15.1)
	Diastolic blood pressure^a^ (mm Hg), mean (SD)		83.1 (11.7)	83.3 (12.0)	82.3 (13.0)	82.4 (12.7)	83.5 (11.8)
	Overweight BMI^b^ (kg/m^2^), n (%)		172 (59.3)	175 (61.4)	161 (56.7)	260 (60.2)	248 (58.1)
**Behavior+lifestyle characteristics**							
	Adequate diet^b^, n (%)		73 (25.2)	75 (26.3)	67 (23.6)	113 (26.2)	102 (23.9)
	Adequate exercise^b^, n (%)		165 (56.9)	150 (52.6)	162 (57)	239 (55.3)	238 (55.7)
	Smokers, n (%)		38 (13.1)	42 (14.7)	35 (12.3)	48 (11.1)	67 (15.7)
**Risk results**							
	Older heart age^c^, n (%)		164 (56.6)	171 (60.0)	153 (53.9)	230 (53.2)	258 (60.4)
	Absolute risk, mean (SD)		5.3 (4.8)	5.4 (4.1)	4.9 (4.1)	4.9 (4.5)	5.5 (4.2)
	Low risk, n (%)		248 (85.5)	235 (82.5)	238 (83.8)	375 (86.8)	346 (81.0)
	Medium risk, n (%)		37 (12.8)	44 (15.4)	39 (13.7)	49 (11.3)	71 (16.6)
	High risk, n (%)		5 (1.7)	6 (2.1)	7 (2.5)	8 (1.9)	10 (2.3)

^a^If known.

^b^Overweight BMI: >25 kg/m^2^; adequate diet: at least 2 servings of fruit and 5 servings of vegetables per day in the past week [51]; adequate physical activity: 3 vigorous sessions per week, 5 moderate sessions per week, or 1-2 vigorous sessions plus 3-4 moderate sessions per week [50].

^c^Older heart age: heart age result is higher than chronological age.

**Table 4:** Trial outcomes by randomized group.

Outcome				Decision aid group						Heart age group		
				Control	Standard		Literacy-sensitive		Risk percentage only			Risk percentage+heart age
**Immediately after the intervention**				(n=290)	(n=285)		(n=284)		(n=432)			(n=427)
		Intention to change lifestyle^a^, mean (SD); 1 (strongly disagree) to 7 (strongly agree)	4.5 (1.4)		4.7 (1.2)	4.6 (1.4)		4.6 (1.3)			4.6 (1.4)	
		Intention to take medication, mean (SD); 1 (strongly disagree) to 7 (strongly agree)	2.5 (1.4)		2.5 (1.4)	2.5 (1.5)		2.5 (1.4)			2.5 (1.4)	
		Intention to take supplements, mean (SD); 1 (strongly disagree) to 7 (strongly agree)	3.2 (1.6)		3.1 (1.6)	3.1 (1.6)		3.1 (1.6)			3.1 (1.6)	
		Decisional conflict, n (%); 4 (yes to all 4 questions; therefore, any score <4 indicates decisional conflict)	34 (11.7)		34 (11.9)	37 (13)		46 (10.6)			59 (13.8)	
		Positive emotion, median (IQR); 0 (none of this feeling) to 10 (a lot of this feeling)	7 (5-8.3)		7.3 (5.3-8.3)	7 (5.3-8.5)		7.3 (5.7-8.7)			6.7 (5-8)	
		Negative emotion, median (IQR); 0 (none of this feeling) to 10 (a lot of this feeling)	1.3 (0-4)		2 (0-5)	2 (0-4.3)		1.2 (0-4)			2 (0-4.7)	
		Credibility, mean (SD); 1 (strongly disagree) to 7 (strongly agree)	5.0 (1.2)		5.0 (1.1)	4.9 (1.2)		5.1 (1.1)			4.9 (1.2)	
		Gist knowledge of risk percentage after the intervention, n (%)	256 (88.3)		253 (88.8)	241 (84.9)		379 (87.7)			371 (86.9)	
		Inflated risk, n (%)	19 (6.6)		16 (5.6)	22 (7.7)		23 (5.3)			34 (8)	
**4-week follow-up (a positive difference means higher levels at follow-up)**				(n=196)	(n=201)		(n=199)		(n=299)			(n=297)
	Difference in smoking^b^, mean (SD)		0.4 (2.1)		−1.4 (7.5)	0.2 (3.2)		0.8 (3.3)			−1.0 (5.7)	
	Difference in moderate exercise^b^, mean (SD)		0.03 (2.2)		−0.1 (2.3)	−0.04 (2.3)		−0.1 (2.3)			0.04 (2.3)	
	Difference in vigorous exercise^b^, mean (SD)		−0.2 (2.2)		−0.1 (2.1)	−0.1 (2.5)		−0.3 (2.1)			0.01 (2.4)	
	Adequate exercise^c^, n (%)		102 (52.0)		103 (51.2)	115 (57.8)		152 (50.8)			169 (56.9)	
	Difference in whether exercise met adequate levels^b^, %		−4.9		−1.4	0.8		−4.5			1.2	
	Difference in daily fruit serves^b^, mean (SD)		−0.4 (2.4)		−0.2 (2.3)	0.5 (2.5)		−0.1 (2.7)			0.01 (2.2)	
	Difference in daily vegetable serves^b^, mean (SD)		−0.4 (2.6)		−0.2 (2.4)	0.1 (2.6)		−0.3 (2.6)			−0.1 (2.5)	
	Difference in daily unhealthy snack serves^b^, mean (SD)		−0.4 (2.2)		−0.3 (2.1)	−0.2 (2.3)		−0.3 (2.2)			−0.4 (2.1)	
	Difference in daily soft drinks^b^, mean (SD)		0.03 (1.6)		−0.1 (1.7)	−0.1 (2.0)		0.1 (1.8)			−0.2 (1.7)	
	Adequate diet^c^, n (%)		39 (19.9)		50 (24.9)	50 (25.1)		68 (22.7)			71 (23.9)	
	Difference in whether diet met adequate levels^b^, %		−5.3		−1.4	1.5		−3.5			0	
	Seen a doctor in the last 4 weeks, n (%)		14 (7.1)		16 (8)	23 (11.6)		27 (9)			26 (8.8)	
	Made an appointment to see a doctor, n (%)		8 (4.1)		7 (3.5)	6 (3)		8 (2.7)			13 (4.4)	
	Called the Heart Foundation helpline in the last 4 weeks, n (%)		1 (0.5)		4 (2)	3 (1.5)		5 (1.7)			3 (1)	
	Gist knowledge of heart age at follow-up, n (%)		44 (22.4)		57 (28.4)	54 (27.1)		40 (13.4)			115 (38.7)	
	Verbatim knowledge of heart age at follow-up, n (%)		16 (8.2)		11 (5.5)	9 (4.5)		2 (0.7)			34 (11.4)	
	Gist knowledge of risk percentage at follow-up, n (%)		76 (38.8)		108 (53.7)	102 (51.3)		139 (46.5)			147 (49.5)	
	Verbatim knowledge of risk percentage at follow-up, n (%)		6 (3.1)		19 (9.5)	14 (7)		21 (7)			18 (6.1)	

^a^Primary outcome.

^b^Difference score: follow-up score minus preintervention score; positive: more at follow-up.

^c^Adequate diet: at least 2 servings of fruit and 5 servings of vegetables per day in the past week [51]; adequate physical activity: 3 vigorous sessions per week, 5 moderate sessions per week, or 1-2 vigorous sessions plus 3-4 moderate sessions per week [50].

**Table 5:** Hypothesis 1: the decision aid (DA) groups will improve outcomes versus the control group.

Outcome		Literacy-sensitive DA vs control				Standard DA vs control				Main effect, *P* value
		Mean difference (95% CI)		*P* value		Mean difference (95% CI)		*P* value		
**Immediately after the intervention**										
	Intention to change lifestyle^a^	0.07 (−0.15 to 0.29)	.52		0.17 (−0.05 to 0.39)		.12		.30	
	Intention to talk to a doctor about medication	0.01 (−0.21 to 0.24)	.90		0.00 (−0.23 to 0.22)		.97		.99	
	Intention to take supplements	−0.10 (−0.36 to 0.16)	.43		−0.09 (−0.34 to 0.17)		.52		.70	
	Decisional conflict^b^	1.12 (0.72 to 1.73)	.62		0.98 (0.63 to 1.54)		.93		.82	
	Positive emotion	0.16 (−0.23 to 0.55)	.43		0.31 (−0.08 to 0.70)		.12		.29	
	Negative emotion	0.28 (−0.11 to 0.68)	.16		0.20 (−0.19 to 0.60)		.31		.34	
	Credibility	−0.12 (−0.30 to 0.07)	.22		0.01 (−0.18 to 0.19)		.95		.34	
	Gist knowledge of risk percentage after the intervention^c^	1.22 (0.74 to 2.02)	.44		1.05 (0.63 to 1.73)		.85		.72	
	Inflated risk^b^	1.10 (0.63 to 1.92)	.74		0.74 (0.39 to 1.38)		.34		.42	
**Follow-up (after 4 weeks, controlling for preintervention)**										
	Daily smoking (number of cigarettes smoked)^d^	0.41 (−2.34 to 3.16)	.77		−1.48 (−4.17 to 1.20)		.28		.29	
	Weekly vigorous exercise sessions^d^	0.23 (−0.15 to 0.62)	.24		0.00 (−0.38 to 0.38)		.99		.39	
	Weekly moderate exercise sessions^d^	0.03 (−0.36 to 0.42)	.89		−0.03 (−0.42 to 0.36)		.87		.95	
	Whether exercise met adequate levels^b^	1.10 (0.95 to 1.28)	.19		1.04 (0.89 to 1.21)		.64		.41	
	Daily fruit serves^d^	0.69 (0.32 to 1.06)	*<*.001		0.21 (−0.13 to 0.55)		.23		<.001	
	Daily vegetable serves^d^	0.38 (−0.03 to 0.78)	.07		0.04 (−0.36 to 0.43)		.85		.13	
	Daily unhealthy snack serves^d^	0.11 (−0.17 to 0.40)	.43		0.02 (−0.26 to 0.31)		.87		.71	
	Daily soft drink serves^d^	0.12 (−0.10 to 0.35)	.28		0.05 (−0.17 to 0.27)		.65		.55	
	Whether diet met adequate levels^b^	1.23 (0.87 to 1.74)	.23		1.16 (0.83 to 1.62)		.37		.48	
**Follow-up only (after 4 weeks)**										
	Has seen a doctor in the last 4 weeks^b^	1.60 (0.85 to 3.02)	.14		1.04 (0.52 to 2.07)		.92		.23	
	Intends to see a doctor at follow-up^b^	0.75 (0.27 to 2.10)	.58		0.86 (0.31 to 2.41)		.78		.86	
	Has called the Heart Foundation helpline in the last 4 weeks^b^	3.00 (0.31 to 29.07)	.34		3.81 (0.45 to 32.25)		.22		.47	
	Gist knowledge of heart age at follow-up^b^	1.12 (0.80 to 1.56)	.51		1.16 (0.84 to 1.61)		.36		.65	
	Verbatim knowledge of heart age at follow-up^b^	0.47 (0.22 to 1.03)	.06		0.58 (0.28 to 1.20)		.14		.12	
	Gist knowledge of risk percentage at follow-up^b^	1.28 (1.04 to 1.58)	.02		1.41 (1.14 to 1.74)		.002		.006	
	Verbatim knowledge of risk percentage at follow-up^b^	2.34 (0.91 to 6.05)	.08		3.25 (1.31 to 8.07)		.01		.04	

^a^Primary outcome.

^b^Analysis by modified Poisson regression, data shown as incidence rate ratios.

^c^Analysis by ordered logistic regression, data shown as odds ratio of being in next highest (relative to group shown Heart Foundation information only).

^d^Analysis by negative binomial regression, data shown as differences in the predicted counts.

**Table 6:** Hypothesis 2: the literacy-sensitive decision aid (DA) will improve outcomes versus the standard DA regardless of health literacy level.

Outcome			Standard DA (vs literacy-sensitive DA)				Newest Vital Signs score×group interaction, *P* value
			Estimated difference (95% CI)		*P* value		
**Immediately after the intervention**							
	Intention to change lifestyle^a^	0.10 (−0.12 to 0.32)		.37		.22	
	Intention to talk to doctor about medication	−0.02 (−0.24 to 0.21)		.87		.02	
	Intention to take supplements	0.02 (−0.24 to 0.28)		.90		.10	
	Decisional conflict^b^	0.88 (0.57 to 1.36)		.56		.53	
	Positive emotion	0.16 (−0.24 to 0.55)		.44		.01	
	Negative emotion	−0.08 (−0.48 to 0.32)		.69		.006	
	Credibility	0.12 (−0.06 to 0.31)		.20		.11	
	Gist knowledge of risk percentage after the intervention^c^	0.86 (0.52 to 1.41)		.55		.007	
	Inflated risk perception (above actual level)^b^	0.70 (0.37 to 1.23)		.20		.72	
**Follow-up (after 4 weeks, controlling for preintervention measurement)**							
	Daily smoking (number of cigarettes smoked)^d^	−1.90 (−4.33 to 0.53)		.13		.90	
	Weekly vigorous exercise sessions^d^	−0.23 (−0.62 to 0.16)		.24		.20	
	Weekly moderate exercise sessions^d^	−0.06 (−0.45 to 0.32)		.76		.50	
	Whether exercise met adequate levels^b^	0.94 (0.81 to 1.09)		.43		.35	
	Daily fruit serves^d^	−0.48 (−0.86 to −0.11)		.01		.15	
	Daily vegetable serves^d^	−0.34 (−0.74 to 0.06)		.10		.10	
	Daily unhealthy snack serves^d^	−0.09 (−0.38 to 0.20)		.53		.97	
	Daily soft drink serves^d^	−0.07 (−0.30 to 0.16)		.53		.77	
	Whether diet met adequate levels^b^	0.94 (0.69 to 1.28)		.71		.90	
**Follow-up (after 4 weeks)**							
	Has seen a doctor in the last 4 weeks^b^	0.65 (0.35 to 1.19)		.16		.75	
	Intends to see a doctor at follow-up^b^	1.15 (0.39 to 3.36)		.80		Not tested (insufficient variability)	
	Has called the Heart Foundation helpline in the last 4 weeks^b^	1.27 (0.26 to 6.09)		.77		<.001	
	Gist knowledge of heart age at follow-up^b^	1.04 (0.77 to 1.41)		.81		.61	
	Verbatim knowledge of heart age at follow-up^b^	1.24 (0.53 to 2.89)		.62		.27	
	Gist knowledge of risk percentage at follow-up^b^	1.10 (0.92 to 1.30)		.29		.83	
	Verbatim knowledge of risk percentage at follow-up^b^	1.39 (0.71 to 2.69)		.33		<.001	

^a^Primary outcome.

^b^Analysis by modified Poisson regression, data shown as incidence rate ratios.

^c^Analysis by ordered logistic regression, data shown as odds ratio of being in next highest (odds in standard, relative to low health literacy).

^d^Analysis by negative binomial regression, data shown as differences in the predicted counts.

**Table 7:** Hypothesis 3: adding heart age to percentage risk will improve outcomes versus percentage risk only.

Outcome			Heart age shown vs not shown																			
			Across all participants						Older heart age result								Same or younger heart age result					
			Estimated mean difference (95% CI)		*P* value			Difference (95% CI)					*P* value			Difference (95% CI)						*P* value
**Immediately after the intervention**																						
	Intention to change lifestyle^a^	−0.04 (−0.22 to 0.14)		.64			−0.11 (−0.34 to 0.13)					.36			0.02 (−0.25 to 0.30)						.87	
	Intention to take medication	.04 (−0.15 to 0.22)		.70			0.11 (−0.13 to 0.36)					.37			−0.14 (−0.41 to 0.13)						.31	
	Intention to take supplements	0.00 (−0.21 to 0.21)		.99			0.07 (−0.21 to 0.35)					.63			−0.14 (−0.47 to 0.19)						.41	
	Decisional conflict^b^	1.27 (0.89 to 1.83)		.19			1.08 (0.72 to 1.62)					.71			1.72 (0.81 to 3.67)						.16	
	Positive emotion	−0.56 (−0.88 to −0.24)		*.*001			−0.75 (−1.19 to −0.31)					*.*001			−0.25 (−0.70 to 0.20)						.28	
	Negative emotion	0.26 (−0.06 to 0.58)		.12			0.57 (0.12 to 1.02)					.01			−0.27 (−0.71 to 0.17)						.23	
	Credibility	−0.20 (−0.35 to −0.05)		**.**01			−0.29 (−0.49 to −0.09)					*.*005			−0.06 (−0.29 to 0.17)						.60	
	Gist knowledge of risk percentage after the intervention^c^	2.03 (1.33 to 3.08)		**.**001			2.12 (1.32 to 3.41)					.002			1.60 (0.58 to 4.37)						.36	
	Inflated risk^b^	1.60 (0.98 to 2.61)		.058			1.70 (0.93 to 3.13)					.09			1.38 (0.57 to 3.36)						.47	
**Follow-up (after 4 weeks, controlling for preintervention measurement)**																						
	Daily smoking (number of cigarettes smoked)^d^	−0.77 (−2.93 to 1.40)				.49	Not estimated (n=4 not shown)			Not estimated				−0.66 (−2.94 to 1.61)						.57		
	Weekly vigorous exercise sessions^d^	0.29 (−0.02 to 0.60)				.07	0.58 (0.09 to 1.07)			.02				0.04 (−0.37 to 0.44)						.85		
	Weekly moderate exercise sessions^d^	0.05 (−0.26 to 0.37)				.74	0.45 (−0.01 to 0.91)			.056				−0.26 (−0.72 to 0.20)						.27		
	Whether exercise met adequate levels^b^	1.16 (0.99 to 1.26)				.08	1.23 (1.05 to 1.45)			.01				1.03 (0.86 to 1.24)						.74		
	Daily fruit serves^d^	0.02 (−0.28 to 0.31)				.92	0.42 (−0.06 to 0.89)			.08				−0.26 (−0.63 to 0.11)						.17		
	Daily vegetable serves^d^	0.30 (−0.02 to 0.63)				.07	0.57 (0.05 to 1.09)			.03				−0.14 (−0.28 to 0.56)						.51		
	Daily unhealthy snack serves^d^	−0.05 (−0.28 to 0.18)				.68	0.22 (−0.15 to 0.58)			.25				−0.28 (−0.58 to 0.02)						.07		
	Daily soft drink serves^d^	−0.14 (−0.33 to 0.04)				.13	0.03 (−0.22 to 0.27)			.83				−0.34 (−0.61 to −0.07)						.01		
	Whether diet met adequate levels^b^	1.14 (0.87 to 1.50)				.34	1.48 (1.00 to 2.18)			.048				0.95 (0.66 to 1.38)						.79		
**Follow-up (after 4 weeks)**																						
	Has seen a doctor in the last 4 weeks^b^	0.99 (0.60 to 1.63)		.96			0.81 (0.37 to 1.80)				.61							1.15 (0.58 to 2.26)	.69			
	Intends to see a doctor at follow-up^b^	1.61 (0.67 to 3.84)		.29			0.67 (0.17 to 0.27)				.58							4.17 (0.90 to 19.32)	.07			
	Has called the Heart Foundation helpline in the last 4 weeks^b^	0.65 (0.17 to 2.53)				.54	1.23 (0.25 to 6.03)			.80				2.66 (1.76 to 4.03)						<.001		
	Gist knowledge of heart age at follow-up^b^	2.90 (2.10 to 3.99)				<.001	3.38 (2.05 to 5.55)			<.001				6.67 (1.50 to 32.41)						.01		
	Verbatim knowledge of heart age at follow-up^b^	18.13 (4.36 to 75.48)				<.001	Not estimated (n=2 not shown)			Not estimated				Not estimated (n=2 not shown)						Not estimated		
	Gist knowledge of risk percentage at follow-up^b^	1.11 (0.95 to 1.30)				.20	1.09 (0.91 to 1.29)			.35				1.16 (0.87 to 1.55)						.31		
	Verbatim knowledge of risk percentage at follow-up^b^	0.82 (0.44 to 1.50)				.52	1.02 (0.40 to 2.57)			.97				0.68 (0.31 to 1.52)						.35		

^a^Primary outcome.

^b^Analysis by modified Poisson regression, data shown as incidence rate ratios.

^c^Analysis by ordered logistic regression, data shown as odds ratio of being in next highest (odds in heart age, relative to not shown).

^d^Analysis by negative binomial regression, data shown as differences in predicted counts and unstable estimate: 1.7% (5/299) individuals who were not shown heart age used the helpline compared with 1.0% (3/297) who were shown heart age.

#### Postintervention Differences Among DA Groups

Immediately after the intervention, there were no differences among the 3 DA groups for the primary outcome of lifestyle intentions or secondary outcomes of risk perception, credibility, emotional response, or decisional conflict. For hypothesis 1, the combined DA groups did not differ from the control group for any outcome (Table 5). For hypothesis 2, there was no difference between standard and literacy-sensitive DAs for any outcome (Table 6). There were significant interactions between DA and health literacy for intention to talk to a doctor about medication (*P=*.02) and emotional responses (positive *P=*.01; negative *P=*.006). Participants with lower health literacy who received literacy-sensitive DA had a more negative or less positive emotional response and had stronger intentions to see a doctor about medication compared with the other groups (Table 6).

#### 4-Week Differences Among DA Groups

At follow-up after 4 weeks, there were no significant differences between the control and DA groups for most self-reported behaviors. However, the literacy-sensitive DA group had higher fruit consumption compared with both the control (difference in predicted counts=0.69, 95% CI 0.32-1.06; *P<*.001) and standard DA groups (difference in predicted counts=0.48, 95% CI 0.11-0.86]; *P=*.01). The DA groups were more likely to know whether their risk was low, medium, or high than the control group (literacy-sensitive DA: incident rate ratio [IRR]**=**1.28, 95% CI 1.04-1.58; *P=*.02 and standard DA: IRR=1.41, 95% CI 1.14-1.74; *P=*.002). The standard DA group was more likely to know their exact risk percentage result compared with the control group (IRR=3.25, 95% CI 1.31-8.07; *P=*.01; Table 5). There were significant differences among DA groups by health literacy levels for self-reported calls to the Heart Foundation helpline (*P<*.001) and verbatim knowledge of CVD percentage risk at follow-up (*P<*.001). None of the participants with low health literacy reported calling the helpline or remembered their exact CVD risk in the control group. Standard DA increased both outcomes in all health literacy groups, and literacy-sensitive DA increased both outcomes in the low and high health literacy groups but not in the medium group (Table 6).

#### Postintervention Differences Among Heart Age Groups

Immediately after the intervention, there were no differences between the 2 heart age groups in the primary outcome of lifestyle intentions or secondary outcomes of risk perception or decisional conflict. For hypothesis 3, the heart age group was less likely to have a positive emotional response (mean difference −0.56, 95% CI −0.88 to −0.24; *P*=.001; Cohen *d*=0.23), less likely to perceive the message as credible (mean difference −0.20, 95% CI −0.35 to −0.05; *P*=.01; Cohen *d*=0.17), and more likely to know whether their risk was low, medium, or high (odds ratio 2.03, 95% CI 1.33-3.08; *P*=.001), compared with the percentage risk only group (Table 7). When the heart age result was older, there were significant differences indicating less positive (mean difference −0.75, 95% CI −1.19 to −0.31; *P*=.001; Cohen *d*=0.31) and more negative (mean difference 0.57, 95% CI 0.12 to 1.02; *P*=.01; Cohen *d*=0.23) emotional responses, lower credibility (mean difference −0.29, 95% CI −0.49 to −0.09; *P*=.005; Cohen *d*=0.25) and higher perceived risk level (odds ratio 2.11, 95% CI 1.31-3.39; *P*=.002) when heart age was shown. No such differences were found in those who received the same age or younger results (Table 7).

#### 4-Week Differences Among Heart Age Groups

At the 4-week follow-up, there were no significant differences among the heart age groups in terms of lifestyle behavior change, seeing a doctor for a heart health check, or gist knowledge of risk level (Table 7). Unsurprisingly, being shown heart age led to greater gist knowledge of heart age (IRR 2.90, 95% CI 2.10-3.99; *P*<.001) and verbatim knowledge of heart age (IRR 18.13, 95% CI 4.36-75.48; *P*<.001) compared with those who were not shown their heart age, but there was no difference between the heart age and percentage risk only groups for knowledge of percentage risk. Within the heart age group that saw both risk formats, participants were more likely to have verbatim knowledge of their heart age (11%) than their percentage risk (6%, chi-square test for paired proportions by McNemar: *χ^2^*_1_=6.1; *P=*.01, difference in proportions 5.4%, 95% CI 0.8%-10.0%). When the heart age result was older, there were significant differences indicating more vigorous exercise (mean difference 0.58, 95% CI 0.09-1.07; *P*=.02), more vegetable serves (mean difference 0.57, 95% CI 0.05-1.09; *P*=.032), higher chance of meeting guidelines for exercise (IRR 1.23, 95% CI 1.05-1.45; *P*=.01) and diet (IRR 1.48, 95% CI 1.00-2.18; *P*=.048), when heart age was shown. When the heart age result was the same or younger than their current age, there were significant differences, indicating fewer soft drink serves (mean difference −0.34, 95% CI −0.61 to −0.07; *P*=.012) and a higher chance of calling the Heart Foundation helpline (IRR 12.66, 95% CI 1.76 to 4.03; *P*<.001), when heart age was shown (Table 7).

### Stage 3

Participant interviews were conducted in 4 stages so that any user feedback from the interviews could be discussed among the team (C Bonner, C Batcup, and JA) and then implemented into the calculator for the next interviews in an iterative process. The issues addressed in each round of interviews are shown in Multimedia Appendix 2.

## Discussion

### Principal Findings

We used both a mixed method development and evaluation process to produce a CVD DA that is effective for improving verbatim and gist knowledge of CVD risk and fruit consumption after 4 weeks. The resulting intervention is a scalable eHealth tool suitable for people with varying levels of health literacy. This consumer tool will supplement a GP version for use within consultations [38,39], providing GPs with a clear action for their patients to follow up when lifestyle change is recommended. This paper provides an example of how to apply literacy-sensitive design principles to evidence-based decision-making and behavior change tools. The results show that literacy-sensitive DAs can support people with lower health literacy in making informed decisions, while still being suitable for the general population.

### Comparison With Previous Work

A recent review of DAs for people with lower health literacy [57,58] showed that DAs that use health literacy design strategies lead to improved knowledge, decisional conflict, and decision-making outcomes. Furthermore, DAs that used explicit strategies to reduce cognitive burden showed greater improvements in knowledge for people with low health literacy and from disadvantaged backgrounds [58]. The review highlighted the need for more consideration of health literacy in DA development. This study addresses these findings in the context of CVD prevention for the first time.

We observed several interactions with health literacy, showing the importance of considering this as a covariate when investigating shared decision-making and behavior change outcomes. The literacy-sensitive version of the DA produced more negative emotional responses and greater intention to speak to a doctor about medication options to reduce CVD risk among those with lower health literacy. This may reflect risk and choice awareness in this group if they had not previously considered themselves to have risk factors for heart disease that could be addressed with preventive medication. As this sample was predominantly low-risk, we would not want a DA to lead to greater actual medication uptake in this group; however, speaking with a physician about risk and how to reduce it may be a positive outcome in line with guidelines to assess risk in this age group [1]. We replicated previous DA studies by finding increased knowledge of risk among the DA groups compared with the control group [37]. We also replicated our previous finding that a literacy-sensitive action plan can improve diet outcomes across different levels of health literacy, although this was more marked for people with low health literacy [31,59].

This study also replicated several heart age effects found in reviews of previous research, in that it leads to a more negative emotional response, increased gist and verbatim knowledge of heart age, but not percentage risk, and reduced credibility, but is neutral for lifestyle change overall [60,61]. Our subgroup analyses suggest that more nuanced study designs are required to better understand the effects of heart age. First, among those who were shown their heart age, gist knowledge of percentage risk initially improved, but after 4 weeks, gist and verbatim knowledge were higher for heart age than for percentage risk. Previous studies have shown that people who receive an older heart age may react defensively and focus on other information, such as a low short-term risk level, which in turn may reduce their credibility of the risk result [26,62]. Analyses of people who received an older heart age result suggest that it may be useful as a marketing tool to gain attention and initiate behavior change, but knowledge of heart age did not translate to knowledge of risk. For the intended purpose of a DA to be used in a clinical context, the focus must be on validated risk results to make informed decisions about medication. Therefore, we decided to use the non–heart age version of the literacy-sensitive DA in future research in general practice. However, web-based heart age tools can incorporate DA and action plan elements with no detrimental effects.

### Future Directions

Future trials need to be designed to isolate older heart age results and follow-up behavior over time. In considering how to power such trials, researchers will need to consider how the specific heart age tool they use is calibrated for the intended population (eg, approximately 50% older in our sample using the New Zealand method vs approximately 80% in the Australian/United Kingdom Heart Foundation tool [25,26]). The primary outcomes also need to be considered carefully. Most heart age research has been conducted with a primary outcome of immediate lifestyle change intentions, where we found no differences. More research could be done to verify the self-reported behavior change among people receiving older heart age results we observed after 4 weeks, using more objective measures such as pedometers.

The end-user interviews were helpful for improving simple navigation and wording issues in the literacy-sensitive version of the DA, but there were some larger issues that could not be resolved using a web-based tool. Most users did not know their blood pressure or cholesterol results; however, even if they had been assessed recently, they had difficulty understanding where different numbers should be entered. This was particularly difficult for cholesterol results in pathology test reports. Therefore, we will test the final revised tool in clinical practice to address the issue of unknown blood pressure and cholesterol, which reduces the accuracy and limits the display of options in line with the current medication guidelines. This tool will be integrated with additional Heart Foundation resources to improve other lifestyle outcomes.

### Strengths and Limitations

A major strength of this study is that we were able to recruit a large, diverse sample in terms of health literacy and risk results. We had sufficient follow-up to run the study per protocol despite the COVID-19 disruptions and observed no difference in dropouts for key variables. A limitation is that the web-based panel sample may not be representative of the general population and may better reflect users of web-based heart age tools than patients presenting to primary care for CVD risk assessment. Furthermore, many participants did not know their blood pressure and cholesterol levels, which may have affected their response to the DA because of a less accurate CVD risk result. However, the use of averages reflects the approach used in currently available consumer tools for CVD risk assessment [26-28]. Different countries also use different CVD risk models or heart age algorithms, which may affect the results given the differences we observed in the older heart age sample. We conducted a large number of analyses on multiple outcomes; however, given the exploratory nature of the study, we did not make adjustments for multiple comparisons. The study was powered by moderate effect sizes and therefore may have lacked the power to detect more subtle differences; however, these findings will be useful for informing sample size calculations for future studies. Finally, we used validated outcomes where possible but behavior changes were self-reported. Future research on heart age should use objective measures over time.

### Conclusions

This study shows the value of combining health-literacy–sensitive design with best practice risk communication and behavior change tools. Although aimed at addressing the needs of people with lower health literacy, this approach improved knowledge of CVD risk, heart age, and behavior in a sample with varying health literacy levels. The role of heart age remains somewhat unclear, with both advantages and disadvantages; however, there is no clear evidence of an effect on lifestyle change intentions or behavior overall. Further research should investigate implementation pathways for integrating such consumer tools with clinical practice and distinguish between older and younger heart age results.

## Appendix

Multimedia Appendix 1 Example intervention content.

Multimedia Appendix 2 Issues addressed in each round of interview feedback.

Multimedia Appendix 3 CONSORT-eHEALTH checklist (V 1.6.1).

## Abbreviations

CONSORT: Consolidated Standards of Reporting Trials
CVD: cardiovascular disease
DA: decision aid
GP: general practitioner
IRR: incident rate ratio
