# Supplementary material for: The Impact of Health Literacy–Sensitive Design and Heart Age in a Cardiovascular Disease Prevention Decision Aid: Randomized Controlled Trial and End-User Testing
Source: JMIR Cardio. 2022 Apr 15;6(1):e34142. doi: 10.2196/34142 (PMC9055529; doi:10.2196/34142)
Supplement: Multimedia Appendix 3 [file cardio_v6i1e34142_app3.pdf]

CONSORT-EHEALTH (V 1.6.1) - Submission/Publication Form

The CONSORT-EHEALTH checklist is intended for authors of randomized trials evaluating web-based and Internet-based applications/interventions, including mobile interventions, electronic games (incl multiplayer games), social media, certain telehealth applications, and other interactive and/or networked electronic applications. Some of the items (e.g. all subitems under item 5 - description of the intervention) may also be applicable for other study designs.

The goal of the CONSORT EHEALTH checklist and guideline is to be
a) a guide for reporting for authors of RCTs,
b) to form a basis for appraisal of an ehealth trial (in terms of validity)

CONSORT-EHEALTH items/subitems are MANDATORY reporting items for studies published in the Journal of Medical Internet Research and other journals / scientific societies endorsing the checklist.

Items numbered 1., 2., 3., 4a., 4b etc are original CONSORT or CONSORT-NPT (non-pharmacologic treatment) items.
Items with Roman numerals (i., ii, iii, iv etc.) are CONSORT-EHEALTH extensions/clarifications.

As the CONSORT-EHEALTH checklist is still considered in a formative stage, we would ask that you also RATE ON A SCALE OF 1-5 how important/useful you feel each item is FOR THE PURPOSE OF THE CHECKLIST and reporting guideline (optional).

Mandatory reporting items are marked with a red *.
In the textboxes, either copy & paste the relevant sections from your manuscript into this form - please include any quotes from your manuscript in QUOTATION MARKS,
or answer directly by providing additional information not in the manuscript, or elaborating on why the item was not relevant for this study.

YOUR ANSWERS WILL BE PUBLISHED AS A SUPPLEMENTARY FILE TO YOUR PUBLICATION IN JMIR AND ARE CONSIDERED PART OF YOUR PUBLICATION (IF ACCEPTED).
Please fill in these questions diligently. Information will not be copyedited, so please use proper spelling and grammar, use correct capitalization, and avoid abbreviations.

DO NOT FORGET TO SAVE AS PDF _AND_ CLICK THE SUBMIT BUTTON SO YOUR ANSWERS ARE IN OUR DATABASE !!!

Citation Suggestion (if you append the pdf as Appendix we suggest to cite this paper in the caption):
Eysenbach G, CONSORT-EHEALTH Group
CONSORT-EHEALTH: Improving and Standardizing Evaluation Reports of Web-based and Mobile Health Interventions
J Med Internet Res 2011;13(4):e126
URL: http://www.jmir.org/2011/4/e126/

doi: 10.2196/jmir.1923
PMID: 22209829

carys.batcup@sydney.edu.au (not shared) Switch accounts

Draft saved

*Required

Your name *

First Last

Carys Batcup

Primary Affiliation (short), City, Country *

University of Toronto, Toronto, Canada

University of Sydney, Sydney, Australia

Your e-mail address *

abc@gmail.com

carys.batcup@sydney.edu.au

Title of your manuscript *

Provide the (draft) title of your manuscript.

The Impact of Health Literacy–Sensitive Design and Heart Age in a Cardiovascular Disease Prevention Decision Aid: Randomized Controlled Trial and End-User Testing

Name of your App/Software/Intervention *

If there is a short and a long/alternate name, write the short name first and add the long name in brackets.

Cardiovascular Disease Prevention Decision A

Evaluated Version (if any)

e.g. "V1", "Release 2017-03-01", "Version 2.0.27913"

Your answer

Language(s) *

What language is the intervention/app in? If multiple languages are available, separate by comma (e.g. "English, French")

English

URL of your Intervention Website or App

e.g. a direct link to the mobile app on app in appstore (itunes, Google Play), or URL of the website. If the intervention is a DVD or hardware, you can also link to an Amazon page.

Your answer

URL of an image/screenshot (optional)

Your answer

Accessibility *

Can an enduser access the intervention presently?

- ○ access is free and open
- ○ access only for special usergroups, not open
- ○ access is open to everyone, but requires payment/subscription/in-app purchases
- ◉ app/intervention no longer accessible
- ○ Other:

Primary Medical Indication/Disease/Condition *

e.g. "Stress", "Diabetes", or define the target group in brackets after the condition, e.g. "Autism (Parents of children with)", "Alzheimers (Informal Caregivers of)"

Cardiovascular disease

Primary Outcomes measured in trial *

comma-separated list of primary outcomes reported in the trial

changing lifestyle intentions or behaviour, incr[illegible]

Secondary/other outcomes

Are there any other outcomes the intervention is expected to affect?

Your answer

Recommended "Dose" *

What do the instructions for users say on how often the app should be used?

- ( ) Approximately Daily
- ( ) Approximately Weekly
- ( ) Approximately Monthly
- ( ) Approximately Yearly
- (x) "as needed"
- ( ) Other:

Approx. Percentage of Users (starters) still using the app as recommended after 3 months *

- (x) unknown / not evaluated
- ( ) 0-10%
- ( ) 11-20%
- ( ) 21-30%
- ( ) 31-40%
- ( ) 41-50%
- ( ) 51-60%
- ( ) 61-70%
- ( ) 71%-80%
- ( ) 81-90%
- ( ) 91-100%
- ( ) Other:

Overall, was the app/intervention effective? *

- ○ yes: all primary outcomes were significantly better in intervention group vs control
- ◉ partly: SOME primary outcomes were significantly better in intervention group vs control
- ○ no statistically significant difference between control and intervention
- ○ potentially harmful: control was significantly better than intervention in one or more outcomes
- ○ inconclusive: more research is needed
- ○ Other:

Article Preparation Status/Stage *

At which stage in your article preparation are you currently (at the time you fill in this form)

- ○ not submitted yet - in early draft status
- ○ not submitted yet - in late draft status, just before submission
- ○ submitted to a journal but not reviewed yet
- ○ submitted to a journal and after receiving initial reviewer comments
- ◉ submitted to a journal and accepted, but not published yet
- ○ published
- ○ Other:

Journal *

If you already know where you will submit this paper (or if it is already submitted), please provide the journal name (if it is not JMIR, provide the journal name under "other")

- ○ not submitted yet / unclear where I will submit this
- ○ Journal of Medical Internet Research (JMIR)
- ○ JMIR mHealth and UHealth
- ○ JMIR Serious Games
- ○ JMIR Mental Health
- ○ JMIR Public Health
- ○ JMIR Formative Research
- ◉ Other JMIR sister journal
- ○ Other:

Is this a full powered effectiveness trial or a pilot/feasibility trial? *

- ○ Pilot/feasibility
- ◉ Fully powered

Manuscript tracking number *

If this is a JMIR submission, please provide the manuscript tracking number under "other" (The ms tracking number can be found in the submission acknowledgement email, or when you login as author in JMIR. If the paper is already published in JMIR, then the ms tracking number is the four-digit number at the end of the DOI, to be found at the bottom of each published article in JMIR)

- ○ no ms number (yet) / not (yet) submitted to / published in JMIR
- ◉ Other: 34142

TITLE AND ABSTRACT

1a) TITLE: Identification as a randomized trial in the title

1a) Does your paper address CONSORT item 1a? *

I.e does the title contain the phrase "Randomized Controlled Trial"? (if not, explain the reason under "other")

- (selected) yes
- Other:

1a-i) Identify the mode of delivery in the title

Identify the mode of delivery. Preferably use “web-based” and/or “mobile” and/or “electronic game” in the title. Avoid ambiguous terms like “online”, “virtual”, “interactive”. Use “Internet-based” only if Intervention includes non-web-based Internet components (e.g. email), use “computer-based” or “electronic” only if offline products are used. Use “virtual” only in the context of “virtual reality” (3-D worlds). Use “online” only in the context of “online support groups”. Complement or substitute product names with broader terms for the class of products (such as “mobile” or “smart phone” instead of “iphone”), especially if the application runs on different platforms.

	1	2	3	4	5	
subitem not at all important	○	○	○	○	○	essential

Does your paper address subitem 1a-i? *

Copy and paste relevant sections from manuscript title (include quotes in quotation marks "like this" to indicate direct quotes from your manuscript), or elaborate on this item by providing additional information not in the ms, or briefly explain why the item is not applicable/relevant for your study

Yes. The mode of delivery was a web-based intervention.

1a-ii) Non-web-based components or important co-interventions in title

Mention non-web-based components or important co-interventions in title, if any (e.g., “with telephone support”).

	1	2	3	4	5	
subitem not at all important	○	○	○	○	○	essential

Does your paper address subitem 1a-ii?

Copy and paste relevant sections from manuscript title (include quotes in quotation marks "like this" to indicate direct quotes from your manuscript), or elaborate on this item by providing additional information not in the ms, or briefly explain why the item is not applicable/relevant for your study

Your answer

1a-iii) Primary condition or target group in the title

Mention primary condition or target group in the title, if any (e.g., “for children with Type I Diabetes”)
Example: A Web-based and Mobile Intervention with Telephone Support for Children with Type I Diabetes: Randomized Controlled Trial

	1	2	3	4	5	
subitem not at all important	○	○	○	○	○	essential

Does your paper address subitem 1a-iii? *

Copy and paste relevant sections from manuscript title (include quotes in quotation marks "like this" to indicate direct quotes from your manuscript), or elaborate on this item by providing additional information not in the ms, or briefly explain why the item is not applicable/relevant for your study

Yes. The target group was people aged 45-74

1b) ABSTRACT: Structured summary of trial design, methods, results, and conclusions

NPT extension: Description of experimental treatment, comparator, care providers, centers, and blinding status.

1b-i) Key features/functionalities/components of the intervention and comparator in the METHODS section of the ABSTRACT

Mention key features/functionalities/components of the intervention and comparator in the abstract. If possible, also mention theories and principles used for designing the site. Keep in mind the needs of systematic reviewers and indexers by including important synonyms. (Note: Only report in the abstract what the main paper is reporting. If this information is missing from the main body of text, consider adding it)

	1	2	3	4	5	
subitem not at all important	○	○	○	○	○	essential

Does your paper address subitem 1b-i? *

Copy and paste relevant sections from the manuscript abstract (include quotes in quotation marks "like this" to indicate direct quotes from your manuscript), or elaborate on this item by providing additional information not in the ms, or briefly explain why the item is not applicable/relevant for your study

Yes. "We developed a standard DA based on international standards. The standard DA was based on our existing general practitioner DA. The literacy-sensitive DA included simple language, supporting images, white space, and a lifestyle action plan. The control DA used Heart Foundation materials."

1b-ii) Level of human involvement in the METHODS section of the ABSTRACT

Clarify the level of human involvement in the abstract, e.g., use phrases like "fully automated" vs. "therapist/nurse/care provider/physician-assisted" (mention number and expertise of providers involved, if any). (Note: Only report in the abstract what the main paper is reporting. If this information is missing from the main body of text, consider adding it)

	1	2	3	4	5	
subitem not at all important	○	○	○	○	○	essential

Does your paper address subitem 1b-ii?

Copy and paste relevant sections from the manuscript abstract (include quotes in quotation marks "like this" to indicate direct quotes from your manuscript), or elaborate on this item by providing additional information not in the ms, or briefly explain why the item is not applicable/relevant for your study

Yes. The level of human involvement was fully automated.

1b-iii) Open vs. closed, web-based (self-assessment) vs. face-to-face assessments in the METHODS section of the ABSTRACT

Mention how participants were recruited (online vs. offline), e.g., from an open access website or from a clinic or a closed online user group (closed usergroup trial), and clarify if this was a purely web-based trial, or there were face-to-face components (as part of the intervention or for assessment). Clearly say if outcomes were self-assessed through questionnaires (as common in web-based trials). Note: In traditional offline trials, an open trial (open-label trial) is a type of clinical trial in which both the researchers and participants know which treatment is being administered. To avoid confusion, use “blinded” or “unblinded” to indicated the level of blinding instead of “open”, as “open” in web-based trials usually refers to “open access” (i.e. participants can self-enrol). (Note: Only report in the abstract what the main paper is reporting. If this information is missing from the main body of text, consider adding it)

	1	2	3	4	5	
subitem not at all important	○	○	○	○	○	essential

Does your paper address subitem 1b-iii?

Copy and paste relevant sections from the manuscript abstract (include quotes in quotation marks "like this" to indicate direct quotes from your manuscript), or elaborate on this item by providing additional information not in the ms, or briefly explain why the item is not applicable/relevant for your study

Yes. Participants were recruited online. This was purely a web-based trial and outcomes were self-assessed through a questionnaire.

1b-iv) RESULTS section in abstract must contain use data

Report number of participants enrolled/assessed in each group, the use/uptake of the intervention (e.g., attrition/adherence metrics, use over time, number of logins etc.), in addition to primary/secondary outcomes. (Note: Only report in the abstract what the main paper is reporting. If this information is missing from the main body of text, consider adding it)

	1	2	3	4	5	
subitem not at all important	○	○	○	○	○	essential

Does your paper address subitem 1b-iv?

Copy and paste relevant sections from the manuscript abstract (include quotes in quotation marks "like this" to indicate direct quotes from your manuscript), or elaborate on this item by providing additional information not in the ms, or briefly explain why the item is not applicable/relevant for your study

Yes, but this is not described in the abstract.

1b-v) CONCLUSIONS/DISCUSSION in abstract for negative trials

Conclusions/Discussions in abstract for negative trials: Discuss the primary outcome - if the trial is negative (primary outcome not changed), and the intervention was not used, discuss whether negative results are attributable to lack of uptake and discuss reasons. (Note: Only report in the abstract what the main paper is reporting. If this information is missing from the main body of text, consider adding it)

	1	2	3	4	5	
subitem not at all important	○	○	○	○	○	essential

Does your paper address subitem 1b-v?

Copy and paste relevant sections from the manuscript abstract (include quotes in quotation marks "like this" to indicate direct quotes from your manuscript), or elaborate on this item by providing additional information not in the ms, or briefly explain why the item is not applicable/relevant for your study

Yes. "The literacy-sensitive DA resulted in increased knowledge of CVD risk and increased fruit consumption in participants with varying health literacy levels and CVD risk results. Adding heart age did not increase lifestyle change intentions or behavior but did affect psychological outcomes, consistent with previous findings"

INTRODUCTION

2a) In INTRODUCTION: Scientific background and explanation of rationale

2a-i) Problem and the type of system/solution

Describe the problem and the type of system/solution that is object of the study: intended as stand-alone intervention vs. incorporated in broader health care program? Intended for a particular patient population? Goals of the intervention, e.g., being more cost-effective to other interventions, replace or complement other solutions? (Note: Details about the intervention are provided in "Methods" under 5)

	1	2	3	4	5	
subitem not at all important	○	○	○	○	○	essential

Does your paper address subitem 2a-i? *

Copy and paste relevant sections from the manuscript (include quotes in quotation marks "like this" to indicate direct quotes from your manuscript), or elaborate on this item by providing additional information not in the ms, or briefly explain why the item is not applicable/relevant for your study

Yes. "There are several evidence-based strategies to address the issue of communicating CVD risk to people with lower health literacy, such as:
1. Use literacy-sensitive design to improve the readability of health information and reduce the cognitive load of action plans for behavior change.
2. Use best practice risk communication formats to explain abstract probabilities (eg, 16%) using icon arrays and more concrete frequencies (eg, 16 out of 100 people like you).
3. Use patient DAs to improve understanding and decision-making, including both lifestyle change and medication, as clear actions that patients can take to reduce their CVD risk.
Objectives
This study aims to develop and test a new consumer engagement tool for CVD prevention based on the aforementioned strategies to address the needs of Australians with different levels of health literacy. It builds on our previous development of a general practitioner (GP)-focused risk calculator and DA and evaluation of the national heart age calculator"

2a-ii) Scientific background, rationale: What is known about the (type of) system

Scientific background, rationale: What is known about the (type of) system that is the object of the study (be sure to discuss the use of similar systems for other conditions/diagnoses, if appropiate), motivation for the study, i.e. what are the reasons for and what is the context for this specific study, from which stakeholder viewpoint is the study performed, potential impact of findings [2]. Briefly justify the choice of the comparator.

	1	2	3	4	5	
subitem not at all important	○	○	○	○	○	essential

Does your paper address subitem 2a-ii? *

Copy and paste relevant sections from the manuscript (include quotes in quotation marks "like this" to indicate direct quotes from your manuscript), or elaborate on this item by providing additional information not in the ms, or briefly explain why the item is not applicable/relevant for your study

Yes. "Prevention of cardiovascular disease (CVD) includes lifestyle interventions and medication for those at highest risk who are most likely to benefit. An absolute risk approach is supported by clinical evidence and endorsed by many national guidelines worldwide. The absolute risk of a heart attack or stroke in the next 5-10 years can be assessed using widely available calculators; however, these tools are substantially underused in practice. Providing medication to high-risk and not low-risk patients is a cost-effective approach. However, up to 75% of high-risk patients do not receive recommended medication to prevent death and disability from CVD, whereas 25% of low-risk patients take medication they are very unlikely to benefit from. Recent guideline changes have led to calls for a shared decision-making approach to ensure that medication prescription for blood pressure and cholesterol is more in line with patient values.
Health literacy also plays a role in CVD prevention. Low health literacy is common in many countries, with estimates ranging from 36% to 60% of the population in Australia, Europe, and the United States. This is associated with poorer self-management, less access to the health system, increased incidence of chronic diseases, including CVD, and increased mortality. Therefore, it is important to engage this group in communication strategies for CVD prevention. This requires changes to the design of web-based patient resources, as many Australians seek health information on the web, but fewer than 1% of health information websites meet the recommended readability levels. Grade 8 is recommended to meet the needs of people with varying health literacy."

2b) In INTRODUCTION: Specific objectives or hypotheses

Does your paper address CONSORT subitem 2b? *

Copy and paste relevant sections from the manuscript (include quotes in quotation marks "like this" to indicate direct quotes from your manuscript), or elaborate on this item by providing additional information not in the ms, or briefly explain why the item is not applicable/relevant for your study

Yes. "This study aims to develop and test a new consumer engagement tool for CVD prevention based on the aforementioned strategies to address the needs of Australians with different levels of health literacy. It builds on our previous development of a general practitioner (GP)-focused risk calculator and DA and evaluation of the national heart age calculator"

METHODS

3a) Description of trial design (such as parallel, factorial) including allocation ratio

Does your paper address CONSORT subitem 3a? *

Copy and paste relevant sections from the manuscript (include quotes in quotation marks "like this" to indicate direct quotes from your manuscript), or elaborate on this item by providing additional information not in the ms, or briefly explain why the item is not applicable/relevant for your study

Yes. "The randomized trial was based on a 3×2 factorial, between-subject design to test the effect of literacy-sensitive design (literacy-sensitive DA, standard DA, or control: Heart Foundation patient information) and risk format (explaining CVD risk only [as a percentage risk], or CVD risk percentage+heart age) on psychological and behavioral outcomes."

3b) Important changes to methods after trial commencement (such as eligibility criteria), with reasons

Does your paper address CONSORT subitem 3b? *

Copy and paste relevant sections from the manuscript (include quotes in quotation marks "like this" to indicate direct quotes from your manuscript), or elaborate on this item by providing additional information not in the ms, or briefly explain why the item is not applicable/relevant for your study

No, as no changes were made after trial commencement.

3b-i) Bug fixes, Downtimes, Content Changes

Bug fixes, Downtimes, Content Changes: ehealth systems are often dynamic systems. A description of changes to methods therefore also includes important changes made on the intervention or comparator during the trial (e.g., major bug fixes or changes in the functionality or content) (5-iii) and other “unexpected events” that may have influenced study design such as staff changes, system failures/downtimes, etc. [2].

	1	2	3	4	5	
subitem not at all important	○	○	○	○	○	essential

Does your paper address subitem 3b-i?

Copy and paste relevant sections from the manuscript (include quotes in quotation marks "like this" to indicate direct quotes from your manuscript), or elaborate on this item by providing additional information not in the ms, or briefly explain why the item is not applicable/relevant for your study

No, as this was a survey so no bug fixes were required.

4a) Eligibility criteria for participants

Does your paper address CONSORT subitem 4a? *

Copy and paste relevant sections from the manuscript (include quotes in quotation marks "like this" to indicate direct quotes from your manuscript), or elaborate on this item by providing additional information not in the ms, or briefly explain why the item is not applicable/relevant for your study

Yes. People aged 45-74 were eligible for inclusion in the study.

4a-i) Computer / Internet literacy

Computer / Internet literacy is often an implicit "de facto" eligibility criterion - this should be explicitly clarified.

	1	2	3	4	5	
subitem not at all important	○	○	○	○	○	essential

Does your paper address subitem 4a-i?

Copy and paste relevant sections from the manuscript (include quotes in quotation marks "like this" to indicate direct quotes from your manuscript), or elaborate on this item by providing additional information not in the ms, or briefly explain why the item is not applicable/relevant for your study

Yes. People in the study could only take part if they were able to access it via the survey software - they had to have signed up to take part in surveys.

4a-ii) Open vs. closed, web-based vs. face-to-face assessments:

Open vs. closed, web-based vs. face-to-face assessments: Mention how participants were recruited (online vs. offline), e.g., from an open access website or from a clinic, and clarify if this was a purely web-based trial, or there were face-to-face components (as part of the intervention or for assessment), i.e., to what degree got the study team to know the participant. In online-only trials, clarify if participants were quasi-anonymous and whether having multiple identities was possible or whether technical or logistical measures (e.g., cookies, email confirmation, phone calls) were used to detect/prevent these.

	1	2	3	4	5	
subitem not at all important	○	○	○	○	○	essential

Does your paper address subitem 4a-ii? *

Copy and paste relevant sections from the manuscript (include quotes in quotation marks "like this" to indicate direct quotes from your manuscript), or elaborate on this item by providing additional information not in the ms, or briefly explain why the item is not applicable/relevant for your study

Yes. Participants were recruited online from a website. This was a purely web-based trial. Participants were anonymous. The survey software has a function which stops people form being able to access the survey more than once.

4a-iii) Information giving during recruitment

Information given during recruitment. Specify how participants were briefed for recruitment and in the informed consent procedures (e.g., publish the informed consent documentation as appendix, see also item X26), as this information may have an effect on user self-selection, user expectation and may also bias results.

	1	2	3	4	5	
subitem not at all important	○	○	○	○	○	essential

Does your paper address subitem 4a-iii?

Copy and paste relevant sections from the manuscript (include quotes in quotation marks "like this" to indicate direct quotes from your manuscript), or elaborate on this item by providing additional information not in the ms, or briefly explain why the item is not applicable/relevant for your study

"A national sample was recruited through Qualtrics (Qualtrics Inc), a web-based social research agency, with stratified sampling based on gender and age groups (5-year age groups from 45 to 74 years). Participants completed a CVD risk assessment based on the Australian guidelines and New Zealand approach to calculate heart age"

4b) Settings and locations where the data were collected

Does your paper address CONSORT subitem 4b? *

Copy and paste relevant sections from the manuscript (include quotes in quotation marks "like this" to indicate direct quotes from your manuscript), or elaborate on this item by providing additional information not in the ms, or briefly explain why the item is not applicable/relevant for your study

Yes. The data was collected online.

4b-i) Report if outcomes were (self-)assessed through online questionnaires

Clearly report if outcomes were (self-)assessed through online questionnaires (as common in web-based trials) or otherwise.

	1	2	3	4	5	
subitem not at all important	○	○	○	○	○	essential

Does your paper address subitem 4b-i? *

Copy and paste relevant sections from the manuscript (include quotes in quotation marks "like this" to indicate direct quotes from your manuscript), or elaborate on this item by providing additional information not in the ms, or briefly explain why the item is not applicable/relevant for your study

Yes. The outcomes were self-assessed through an online questionnaire.

4b-ii) Report how institutional affiliations are displayed

Report how institutional affiliations are displayed to potential participants [on ehealth media], as affiliations with prestigious hospitals or universities may affect volunteer rates, use, and reactions with regards to an intervention.(Not a required item – describe only if this may bias results)

	1	2	3	4	5	
subitem not at all important	○	○	○	○	○	essential

Does your paper address subitem 4b-ii?

Copy and paste relevant sections from the manuscript (include quotes in quotation marks "like this" to indicate direct quotes from your manuscript), or elaborate on this item by providing additional information not in the ms, or briefly explain why the item is not applicable/relevant for your study

Your answer

5) The interventions for each group with sufficient details to allow replication, including how and when they were actually administered

5-i) Mention names, credential, affiliations of the developers, sponsors, and owners

Mention names, credential, affiliations of the developers, sponsors, and owners [6] (if authors/evaluators are owners or developer of the software, this needs to be declared in a “Conflict of interest” section or mentioned elsewhere in the manuscript).

	1	2	3	4	5	
subitem not at all important	○	○	○	○	○	essential

Does your paper address subitem 5-i?

Copy and paste relevant sections from the manuscript (include quotes in quotation marks "like this" to indicate direct quotes from your manuscript), or elaborate on this item by providing additional information not in the ms, or briefly explain why the item is not applicable/relevant for your study

No this is not relevant to this study as it's just a survey, not something that will be relevant again. However we are working on an updated tool that may be integrated into general practice.

5-ii) Describe the history/development process

Describe the history/development process of the application and previous formative evaluations (e.g., focus groups, usability testing), as these will have an impact on adoption/use rates and help with interpreting results.

	1	2	3	4	5	
subitem not at all important	○	○	○	○	○	essential

Does your paper address subitem 5-ii?

Copy and paste relevant sections from the manuscript (include quotes in quotation marks "like this" to indicate direct quotes from your manuscript), or elaborate on this item by providing additional information not in the ms, or briefly explain why the item is not applicable/relevant for your study

Yes. "In stage one, we developed a literacy-sensitive version of our existing GP DA, which calculates 5-year risk of a CVD event based on current guidelines and shows the effects of 9 lifestyle, medication, and supplement interventions. This was based on previous reviews and evaluations of 73 CVD risk calculators and 25 CVD prevention DAs, which identified tools for many different CVD models, but none that matched Australian guidelines and best practice communication principles. We added heart age to the Australian absolute CVD risk calculation based on published methods from New Zealand, both of which use the 5-year Framingham equation. The literacy-sensitive design included simple language, supporting images, and white space to improve readability and understandability. The text within this DA was evaluated using the Sydney Health Literacy Editor, a tool that automatically applies readability and actionability criteria to the text. On the basis of this feedback, the final tool met the recommended grade 8 level. The literacy-sensitive version also included a novel action plan format developed by our team, which has been shown to reduce unhealthy lifestyle behaviors among people with low health literacy. We added options for physical activity and smoking to the existing tools to reduce unhealthy snacking, drawing on previous literature on effective if-then plans in these areas. If-then plans help people identify an important environment context or trigger in which they find that they often carry out an unwanted behavior and to identify a new behavior that can be substituted for the unwanted behavior. These 2 components are formulated into an if-then statement or plan; for example, If I find myself eating unhealthy snacks when drinking a cup of tea, then I will eat a piece of fruit instead. In this study, we used an if-then format called a volitional help sheet, which prompts the person with predefined if and then statements."

5-iii) Revisions and updating

Revisions and updating. Clearly mention the date and/or version number of the application/intervention (and comparator, if applicable) evaluated, or describe whether the intervention underwent major changes during the evaluation process, or whether the development and/or content was "frozen" during the trial. Describe dynamic components such as news feeds or changing content which may have an impact on the replicability of the intervention (for unexpected events see item 3b).

	1	2	3	4	5	
subitem not at all important	○	○	○	○	○	essential

Does your paper address subitem 5-iii?

Copy and paste relevant sections from the manuscript (include quotes in quotation marks "like this" to indicate direct quotes from your manuscript), or elaborate on this item by providing additional information not in the ms, or briefly explain why the item is not applicable/relevant for your study

No, this is not relevant for this paper. However, user testing was completed at the end (after the trial took place)

5-iv) Quality assurance methods

Provide information on quality assurance methods to ensure accuracy and quality of information provided [1], if applicable.

	1	2	3	4	5	
subitem not at all important	○	○	○	○	○	essential

Does your paper address subitem 5-iv?

Copy and paste relevant sections from the manuscript (include quotes in quotation marks "like this" to indicate direct quotes from your manuscript), or elaborate on this item by providing additional information not in the ms, or briefly explain why the item is not applicable/relevant for your study

Yes. "the Australian absolute CVD risk calculation based on published methods from New Zealand, both of which use the 5-year Framingham equation."

5-v) Ensure replicability by publishing the source code, and/or providing screenshots/screen-capture video, and/or providing flowcharts of the algorithms used

Ensure replicability by publishing the source code, and/or providing screenshots/screen-capture video, and/or providing flowcharts of the algorithms used. Replicability (i.e., other researchers should in principle be able to replicate the study) is a hallmark of scientific reporting.

	1	2	3	4	5	
subitem not at all important	○	○	○	○	○	essential

Does your paper address subitem 5-v?

Copy and paste relevant sections from the manuscript (include quotes in quotation marks "like this" to indicate direct quotes from your manuscript), or elaborate on this item by providing additional information not in the ms, or briefly explain why the item is not applicable/relevant for your study

No this is not relevant for this paper as the tool was just used in this survey.

5-vi) Digital preservation

Digital preservation: Provide the URL of the application, but as the intervention is likely to change or disappear over the course of the years; also make sure the intervention is archived (Internet Archive, webcitation.org, and/or publishing the source code or screenshots/videos alongside the article). As pages behind login screens cannot be archived, consider creating demo pages which are accessible without login.

	1	2	3	4	5	
subitem not at all important	○	○	○	○	○	essential

Does your paper address subitem 5-vi?

Copy and paste relevant sections from the manuscript (include quotes in quotation marks "like this" to indicate direct quotes from your manuscript), or elaborate on this item by providing additional information not in the ms, or briefly explain why the item is not applicable/relevant for your study

No, however the URL of our other tool is provided (auscvdrisk.com.au)

5-vii) Access

Access: Describe how participants accessed the application, in what setting/context, if they had to pay (or were paid) or not, whether they had to be a member of specific group. If known, describe how participants obtained “access to the platform and Internet” [1]. To ensure access for editors/reviewers/readers, consider to provide a “backdoor” login account or demo mode for reviewers/readers to explore the application (also important for archiving purposes, see vi).

	1	2	3	4	5	
subitem not at all important	○	○	○	○	○	essential

Does your paper address subitem 5-vii? *

Copy and paste relevant sections from the manuscript (include quotes in quotation marks "like this" to indicate direct quotes from your manuscript), or elaborate on this item by providing additional information not in the ms, or briefly explain why the item is not applicable/relevant for your study

No, this is not relevant as it was a survey that participants were sent.

5-viii) Mode of delivery, features/functionalities/components of the intervention and comparator, and the theoretical framework

Describe mode of delivery, features/functionalities/components of the intervention and comparator, and the theoretical framework [6] used to design them (instructional strategy [1], behaviour change techniques, persuasive features, etc., see e.g., [7, 8] for terminology). This includes an in-depth description of the content (including where it is coming from and who developed it) [1],” whether [and how] it is tailored to individual circumstances and allows users to track their progress and receive feedback” [6]. This also includes a description of communication delivery channels and – if computer-mediated communication is a component – whether communication was synchronous or asynchronous [6]. It also includes information on presentation strategies [1], including page design principles, average amount of text on pages, presence of hyperlinks to other resources, etc. [1].

	1	2	3	4	5	
subitem not at all important	○	○	○	○	○	essential

Does your paper address subitem 5-viii? *

Copy and paste relevant sections from the manuscript (include quotes in quotation marks "like this" to indicate direct quotes from your manuscript), or elaborate on this item by providing additional information not in the ms, or briefly explain why the item is not applicable/relevant for your study

Yes. "In stage one, we developed a literacy-sensitive version of our existing GP DA, which calculates 5-year risk of a CVD event based on current guidelines and shows the effects of 9 lifestyle, medication, and supplement interventions. This was based on previous reviews and evaluations of 73 CVD risk calculators and 25 CVD prevention DAs, which identified tools for many different CVD models, but none that matched Australian guidelines and best practice communication principles. We added heart age to the Australian absolute CVD risk calculation based on published methods from New Zealand, both of which use the 5-year Framingham equation. The literacy-sensitive design included simple language, supporting images, and white space to improve readability and understandability. The text within this DA was evaluated using the Sydney Health Literacy Editor, a tool that automatically applies readability and actionability criteria to the text. On the basis of this feedback, the final tool met the recommended grade 8 level. The literacy-sensitive version also included a novel action plan format developed by our team, which has been shown to reduce unhealthy lifestyle behaviors among people with low health literacy. We added options for physical activity and smoking to the existing tools to reduce unhealthy snacking, drawing on previous literature on effective if-then plans in these areas. If-then plans help people identify an important environment context or trigger in which they find that they often carry out an unwanted behavior and to identify a new behavior that can be substituted for the unwanted behavior. These 2 components are formulated into an if-then statement or plan; for example, If I find myself eating unhealthy snacks when drinking a cup of tea, then I will eat a piece of fruit instead. In this study, we used an if-then format called a volitional help sheet, which prompts the person with predefined if and then statements."

5-ix) Describe use parameters

Describe use parameters (e.g., intended “doses” and optimal timing for use). Clarify what instructions or recommendations were given to the user, e.g., regarding timing, frequency, heaviness of use, if any, or was the intervention used ad libitum.

	1	2	3	4	5	
subitem not at all important	○	○	○	○	○	essential

Does your paper address subitem 5-ix?

Copy and paste relevant sections from the manuscript (include quotes in quotation marks "like this" to indicate direct quotes from your manuscript), or elaborate on this item by providing additional information not in the ms, or briefly explain why the item is not applicable/relevant for your study

No, this is not relevant for this study as participants simply went through the survey once.

5-x) Clarify the level of human involvement

Clarify the level of human involvement (care providers or health professionals, also technical assistance) in the e-intervention or as co-intervention (detail number and expertise of professionals involved, if any, as well as “type of assistance offered, the timing and frequency of the support, how it is initiated, and the medium by which the assistance is delivered”. It may be necessary to distinguish between the level of human involvement required for the trial, and the level of human involvement required for a routine application outside of a RCT setting (discuss under item 21 – generalizability).

	1	2	3	4	5	
subitem not at all important	○	○	○	○	○	essential

Does your paper address subitem 5-x?

Copy and paste relevant sections from the manuscript (include quotes in quotation marks "like this" to indicate direct quotes from your manuscript), or elaborate on this item by providing additional information not in the ms, or briefly explain why the item is not applicable/relevant for your study

Yes, this survey required no help, purely self-assessed.

5-xi) Report any prompts/reminders used

Report any prompts/reminders used: Clarify if there were prompts (letters, emails, phone calls, SMS) to use the application, what triggered them, frequency etc. It may be necessary to distinguish between the level of prompts/reminders required for the trial, and the level of prompts/reminders for a routine application outside of a RCT setting (discuss under item 21 – generalizability).

	1	2	3	4	5	
subitem not at all important	○	○	○	○	○	essential

Does your paper address subitem 5-xi? *

Copy and paste relevant sections from the manuscript (include quotes in quotation marks "like this" to indicate direct quotes from your manuscript), or elaborate on this item by providing additional information not in the ms, or briefly explain why the item is not applicable/relevant for your study

Yes. Participants were required to do one survey and then asked to do a follow up survey after 4 weeks.

5-xii) Describe any co-interventions (incl. training/support)

Describe any co-interventions (incl. training/support): Clearly state any interventions that are provided in addition to the targeted eHealth intervention, as ehealth intervention may not be designed as stand-alone intervention. This includes training sessions and support [1]. It may be necessary to distinguish between the level of training required for the trial, and the level of training for a routine application outside of a RCT setting (discuss under item 21 – generalizability.

	1	2	3	4	5	
subitem not at all important	○	○	○	○	○	essential

Does your paper address subitem 5-xii? *

Copy and paste relevant sections from the manuscript (include quotes in quotation marks "like this" to indicate direct quotes from your manuscript), or elaborate on this item by providing additional information not in the ms, or briefly explain why the item is not applicable/relevant for your study

No, as it's not relevant. No support was required.

6a) Completely defined pre-specified primary and secondary outcome measures, including how and when they were assessed

Does your paper address CONSORT subitem 6a? *

Copy and paste relevant sections from the manuscript (include quotes in quotation marks "like this" to indicate direct quotes from your manuscript), or elaborate on this item by providing additional information not in the ms, or briefly explain why the item is not applicable/relevant for your study

Yes. "Established measures were used for the primary outcome of behavioral intentions (validated theory of planned behavior scale applied to smoking, diet, exercise, and GP visit). Secondary outcomes included self-reported behavior after 4 weeks compared with national guidelines for diet and physical activity, gist and verbatim knowledge (absolute risk percentage and heart age), emotional response using a validated scale (3 positive emotions, eg, hopeful, and 3 negative emotions, eg, anxious), credibility of the information (that the information is personally relevant), and decision conflict scale (uncertainty in decision-making)"

6a-i) Online questionnaires: describe if they were validated for online use and apply CHERRIES items to describe how the questionnaires were designed/deployed

If outcomes were obtained through online questionnaires, describe if they were validated for online use and apply CHERRIES items to describe how the questionnaires were designed/deployed [9].

	1	2	3	4	5	
subitem not at all important	○	○	○	○	○	essential

Does your paper address subitem 6a-i?

Copy and paste relevant sections from manuscript text

Yes. We used established measures for the questionnaire items.

6a-ii) Describe whether and how “use” (including intensity of use/dosage) was defined/measured/monitored

Describe whether and how “use” (including intensity of use/dosage) was defined/measured/monitored (logins, logfile analysis, etc.). Use/adoption metrics are important process outcomes that should be reported in any ehealth trial.

	1	2	3	4	5	
subitem not at all important	○	○	○	○	○	essential

Does your paper address subitem 6a-ii?

Copy and paste relevant sections from manuscript text

No, as participants were not required to use the tool more than once.

6a-iii) Describe whether, how, and when qualitative feedback from participants was obtained

Describe whether, how, and when qualitative feedback from participants was obtained (e.g., through emails, feedback forms, interviews, focus groups).

	1	2	3	4	5	
subitem not at all important	○	○	○	○	○	essential

Does your paper address subitem 6a-iii?

Copy and paste relevant sections from manuscript text

Yes. "As part of the follow-up survey, participants in the trial were invited to opt-in to a think aloud interview to provide further end-user testing and feedback for the literacy-sensitive version of the intervention. From the 27 participants who provided email addresses, 20 (74%) participants were selected to represent a range of ages, genders, risk levels, and health literacy levels. Participants went through the risk calculator in full while saying out loud everything they were thinking; for example, any areas of confusion. Further questions were asked to prompt more discussion or elaboration. Transcripts were thematically coded and discussed after each set of 4-5 interviews, and improvements were made to the intervention before the next set of interviews. We conducted 2 rounds of interviews with people with low health literacy as our key target group (8/20, 40%) and then tested the improved tool with people who had higher health literacy to ensure that it was suitable for these users in another 2 rounds (12/20, 60%)."

6b) Any changes to trial outcomes after the trial commenced, with reasons

Does your paper address CONSORT subitem 6b? *

Copy and paste relevant sections from the manuscript (include quotes in quotation marks "like this" to indicate direct quotes from your manuscript), or elaborate on this item by providing additional information not in the ms, or briefly explain why the item is not applicable/relevant for your study

No, as no changes were made.

7a) How sample size was determined

NPT: When applicable, details of whether and how the clustering by care provides or centers was addressed

7a-i) Describe whether and how expected attrition was taken into account when calculating the sample size

Describe whether and how expected attrition was taken into account when calculating the sample size.

	1	2	3	4	5	
subitem not at all important	○	○	○	○	○	essential

Does your paper address subitem 7a-i?

Copy and paste relevant sections from manuscript title (include quotes in quotation marks "like this" to indicate direct quotes from your manuscript), or elaborate on this item by providing additional information not in the ms, or briefly explain why the item is not applicable/relevant for your study

Yes. "A priori sample size calculations determined that 85 participants per randomized group (total n=510) would yield 90% power to detect a moderate effect size of Cohen d=0.5 (a standardized difference; this generic effect size estimate was selected because of the absence of similar trials on which to base calculations) in the primary outcome of intention to change lifestyle or any of the secondary outcomes, assuming a 2-sided Cronbach α of .05. We aimed to recruit an additional 20% of cases to account for potential missing values, totaling 600 participants (100 per group) at follow-up. This sample was inflated for recruitment to 850 participants to account for potential attrition of up to 30% between the intervention and follow-up."

7b) When applicable, explanation of any interim analyses and stopping guidelines

Does your paper address CONSORT subitem 7b? *

Copy and paste relevant sections from the manuscript (include quotes in quotation marks "like this" to indicate direct quotes from your manuscript), or elaborate on this item by providing additional information not in the ms, or briefly explain why the item is not applicable/relevant for your study

No, as this is not relevant to this study.

8a) Method used to generate the random allocation sequence

NPT: When applicable, how care providers were allocated to each trial group

Does your paper address CONSORT subitem 8a? *

Copy and paste relevant sections from the manuscript (include quotes in quotation marks "like this" to indicate direct quotes from your manuscript), or elaborate on this item by providing additional information not in the ms, or briefly explain why the item is not applicable/relevant for your study

No, but this is demonstrated in Figure 3.

8b) Type of randomisation; details of any restriction (such as blocking and block size)

Does your paper address CONSORT subitem 8b? *

Copy and paste relevant sections from the manuscript (include quotes in quotation marks "like this" to indicate direct quotes from your manuscript), or elaborate on this item by providing additional information not in the ms, or briefly explain why the item is not applicable/relevant for your study

No, as no restrictions to randomisation were included.

9) Mechanism used to implement the random allocation sequence (such as sequentially numbered containers), describing any steps taken to conceal the sequence until interventions were assigned

Does your paper address CONSORT subitem 9? *

Copy and paste relevant sections from the manuscript (include quotes in quotation marks "like this" to indicate direct quotes from your manuscript), or elaborate on this item by providing additional information not in the ms, or briefly explain why the item is not applicable/relevant for your study

No, as randomisation was done automatically by the survey software.

10) Who generated the random allocation sequence, who enrolled participants, and who assigned participants to interventions

Does your paper address CONSORT subitem 10? *

Copy and paste relevant sections from the manuscript (include quotes in quotation marks "like this" to indicate direct quotes from your manuscript), or elaborate on this item by providing additional information not in the ms, or briefly explain why the item is not applicable/relevant for your study

No, as randomisation was done automatically by the survey software.

11a) If done, who was blinded after assignment to interventions (for example, participants, care providers, those assessing outcomes) and how

NPT: Whether or not administering co-interventions were blinded to group assignment

11a-i) Specify who was blinded, and who wasn’t

Specify who was blinded, and who wasn’t. Usually, in web-based trials it is not possible to blind the participants [1, 3] (this should be clearly acknowledged), but it may be possible to blind outcome assessors, those doing data analysis or those administering co-interventions (if any).

	1	2	3	4	5	
subitem not at all important	○	○	○	○	○	essential

Does your paper address subitem 11a-i? *

Copy and paste relevant sections from the manuscript (include quotes in quotation marks "like this" to indicate direct quotes from your manuscript), or elaborate on this item by providing additional information not in the ms, or briefly explain why the item is not applicable/relevant for your study

No, as randomisation was done automatically by the survey software.

11a-ii) Discuss e.g., whether participants knew which intervention was the “intervention of interest” and which one was the “comparator”

Informed consent procedures (4a-ii) can create biases and certain expectations - discuss e.g., whether participants knew which intervention was the “intervention of interest” and which one was the “comparator”.

	1	2	3	4	5	
subitem not at all important	○	○	○	○	○	essential

Does your paper address subitem 11a-ii?

Copy and paste relevant sections from the manuscript (include quotes in quotation marks "like this" to indicate direct quotes from your manuscript), or elaborate on this item by providing additional information not in the ms, or briefly explain why the item is not applicable/relevant for your study

No, as randomisation was done automatically by the survey software, and participants weren't informed of this beforehand.

11b) If relevant, description of the similarity of interventions

(this item is usually not relevant for ehealth trials as it refers to similarity of a placebo or sham intervention to a active medication/intervention)

Does your paper address CONSORT subitem 11b? *

Copy and paste relevant sections from the manuscript (include quotes in quotation marks "like this" to indicate direct quotes from your manuscript), or elaborate on this item by providing additional information not in the ms, or briefly explain why the item is not applicable/relevant for your study

Yes. See Table 1 and Figure 1 for intervention descriptions and examples.

12a) Statistical methods used to compare groups for primary and secondary outcomes

NPT: When applicable, details of whether and how the clustering by care providers or centers was addressed

Does your paper address CONSORT subitem 12a? *

Copy and paste relevant sections from the manuscript (include quotes in quotation marks "like this" to indicate direct quotes from your manuscript), or elaborate on this item by providing additional information not in the ms, or briefly explain why the item is not applicable/relevant for your study

Yes. "Continuous outcome variables were modeled using linear regression. Dichotomous outcomes were analyzed using modified Poisson regression (using a log-link function with robust error variances). Ordinal logistic regression was used to analyze the ordered categorical outcomes. Count outcomes were modeled using negative binomial regression. All regression models included the DA group (literacy-sensitive DA, standard DA, or basic Heart Foundation patient information) and risk format (CVD risk percentage only or CVD risk percentage+heart age) as categorical variables and controlled for health literacy adequacy (categorical based on the Newest Vital Signs measure: low, moderate, or adequate) and absolute risk (percentage). Postintervention and follow-up outcomes were analyzed separately, with follow-up analyses controlling for preintervention values where available. Pairwise comparisons were conducted to test these hypotheses. We also conducted exploratory analyses of potential differences in DA effects between health literacy levels by including a literacy-sensitive-by-DA interaction term and heart age category for heart age groups (younger or same vs older in stratified analyses). Chi-square test for paired proportions by McNemar was used to compare knowledge of heart age versus percentage risk among those who saw both. Analyses were conducted using Stata (version 16.1; StataCorp). No adjustments were made for multiple comparisons."

12a-i) Imputation techniques to deal with attrition / missing values

Imputation techniques to deal with attrition / missing values: Not all participants will use the intervention/comparator as intended and attrition is typically high in ehealth trials. Specify how participants who did not use the application or dropped out from the trial were treated in the statistical analysis (a complete case analysis is strongly discouraged, and simple imputation techniques such as LOCF may also be problematic [4]).

	1	2	3	4	5	
subitem not at all important	○	○	○	○	○	essential

Does your paper address subitem 12a-i? *

Copy and paste relevant sections from the manuscript (include quotes in quotation marks "like this" to indicate direct quotes from your manuscript), or elaborate on this item by providing additional information not in the ms, or briefly explain why the item is not applicable/relevant for your study

Yes. "We aimed to recruit an additional 20% of cases to account for potential missing values, totaling 600 participants (100 per group) at follow-up. This sample was inflated for recruitment to 850 participants to account for potential attrition of up to 30% between the intervention and follow-up."

12b) Methods for additional analyses, such as subgroup analyses and adjusted analyses

Does your paper address CONSORT subitem 12b? *

Copy and paste relevant sections from the manuscript (include quotes in quotation marks "like this" to indicate direct quotes from your manuscript), or elaborate on this item by providing additional information not in the ms, or briefly explain why the item is not applicable/relevant for your study

Yes. "Continuous outcome variables were modeled using linear regression. Dichotomous outcomes were analyzed using modified Poisson regression (using a log-link function with robust error variances). Ordinal logistic regression was used to analyze the ordered categorical outcomes. Count outcomes were modeled using negative binomial regression. All regression models included the DA group (literacy-sensitive DA, standard DA, or basic Heart Foundation patient information) and risk format (CVD risk percentage only or CVD risk percentage+heart age) as categorical variables and controlled for health literacy adequacy (categorical based on the Newest Vital Signs measure: low, moderate, or adequate) and absolute risk (percentage). Postintervention and follow-up outcomes were analyzed separately, with follow-up analyses controlling for preintervention values where available. Pairwise comparisons were conducted to test these hypotheses. We also conducted exploratory analyses of potential differences in DA effects between health literacy levels by including a literacy-sensitive-by-DA interaction term and heart age category for heart age groups (younger or same vs older in stratified analyses). Chi-square test for paired proportions by McNemar was used to compare knowledge of heart age versus percentage risk among those who saw both. Analyses were conducted using Stata (version 16.1; StataCorp). No adjustments were made for multiple comparisons."

X26) REB/IRB Approval and Ethical Considerations [recommended as subheading under "Methods"] (not a CONSORT item)

X26-i) Comment on ethics committee approval

	1	2	3	4	5	
subitem not at all important	○	○	○	○	○	essential

Does your paper address subitem X26-i?

Copy and paste relevant sections from the manuscript (include quotes in quotation marks "like this" to indicate direct quotes from your manuscript), or elaborate on this item by providing additional information not in the ms, or briefly explain why the item is not applicable/relevant for your study

Yes. "This study received ethics approval from the Human Research Ethics Committee of the University of Sydney (project number 2019/774)."

x26-ii) Outline informed consent procedures

Outline informed consent procedures e.g., if consent was obtained offline or online (how? Checkbox, etc.?), and what information was provided (see 4a-ii). See [6] for some items to be included in informed consent documents.

	1	2	3	4	5	
subitem not at all important	○	○	○	○	○	essential

Does your paper address subitem X26-ii?

Copy and paste relevant sections from the manuscript (include quotes in quotation marks "like this" to indicate direct quotes from your manuscript), or elaborate on this item by providing additional information not in the ms, or briefly explain why the item is not applicable/relevant for your study

No, we have not included the participant information sheet, however participants were shown this and asked to consent to taking part. As shown in Figure 3, the CONSORT diagram, 211 participants did not consent to take part.

X26-iii) Safety and security procedures

Safety and security procedures, incl. privacy considerations, and any steps taken to reduce the likelihood or detection of harm (e.g., education and training, availability of a hotline)

	1	2	3	4	5	
subitem not at all important	○	○	○	○	○	essential

Does your paper address subitem X26-iii?

Copy and paste relevant sections from the manuscript (include quotes in quotation marks "like this" to indicate direct quotes from your manuscript), or elaborate on this item by providing additional information not in the ms, or briefly explain why the item is not applicable/relevant for your study

Your answer

RESULTS

13a) For each group, the numbers of participants who were randomly assigned, received intended treatment, and were analysed for the primary outcome

NPT: The number of care providers or centers performing the intervention in each group and the number of patients treated by each care provider in each center

Does your paper address CONSORT subitem 13a? *

Copy and paste relevant sections from the manuscript (include quotes in quotation marks "like this" to indicate direct quotes from your manuscript), or elaborate on this item by providing additional information not in the ms, or briefly explain why the item is not applicable/relevant for your study

Yes, the CONSORT diagram (Figure 3) shows this.

13b) For each group, losses and exclusions after randomisation, together with reasons

Does your paper address CONSORT subitem 13b? (NOTE: Preferably, this is shown in a CONSORT flow diagram) *

Copy and paste relevant sections from the manuscript (include quotes in quotation marks "like this" to indicate direct quotes from your manuscript), or elaborate on this item by providing additional information not in the ms, or briefly explain why the item is not applicable/relevant for your study

Yes, the CONSORT diagram (Figure 3) shows this.

13b-i) Attrition diagram

Strongly recommended: An attrition diagram (e.g., proportion of participants still logging in or using the intervention/comparator in each group plotted over time, similar to a survival curve) or other figures or tables demonstrating usage/dose/engagement.

	1	2	3	4	5	
subitem not at all important	○	○	○	○	○	essential

Does your paper address subitem 13b-i?

Copy and paste relevant sections from the manuscript or cite the figure number if applicable (include quotes in quotation marks "like this" to indicate direct quotes from your manuscript), or elaborate on this item by providing additional information not in the ms, or briefly explain why the item is not applicable/relevant for your study

No, as this was a one-off intervention

14a) Dates defining the periods of recruitment and follow-up

Does your paper address CONSORT subitem 14a? *

Copy and paste relevant sections from the manuscript (include quotes in quotation marks "like this" to indicate direct quotes from your manuscript), or elaborate on this item by providing additional information not in the ms, or briefly explain why the item is not applicable/relevant for your study

No, as the dates are not relevant. However the paper is clear that the follow up survey was sent 4 weeks after the initial survey.

14a-i) Indicate if critical “secular events” fell into the study period

Indicate if critical “secular events” fell into the study period, e.g., significant changes in Internet resources available or “changes in computer hardware or Internet delivery resources”

	1	2	3	4	5	
subitem not at all important	○	○	○	○	○	essential

Does your paper address subitem 14a-i?

Copy and paste relevant sections from the manuscript (include quotes in quotation marks "like this" to indicate direct quotes from your manuscript), or elaborate on this item by providing additional information not in the ms, or briefly explain why the item is not applicable/relevant for your study

Your answer

14b) Why the trial ended or was stopped (early)

Does your paper address CONSORT subitem 14b? *

Copy and paste relevant sections from the manuscript (include quotes in quotation marks "like this" to indicate direct quotes from your manuscript), or elaborate on this item by providing additional information not in the ms, or briefly explain why the item is not applicable/relevant for your study

No, as there was no end date, just when we completed recruitment for the total number of participants.

15) A table showing baseline demographic and clinical characteristics for each group

NPT: When applicable, a description of care providers (case volume, qualification, expertise, etc.) and centers (volume) in each group

Does your paper address CONSORT subitem 15? *

Copy and paste relevant sections from the manuscript (include quotes in quotation marks "like this" to indicate direct quotes from your manuscript), or elaborate on this item by providing additional information not in the ms, or briefly explain why the item is not applicable/relevant for your study

Yes. This is Table 3 in the paper.

15-i) Report demographics associated with digital divide issues

In ehealth trials it is particularly important to report demographics associated with digital divide issues, such as age, education, gender, social-economic status, computer/Internet/ehealth literacy of the participants, if known.

	1	2	3	4	5	
subitem not at all important	○	○	○	○	○	essential

Does your paper address subitem 15-i? *

Copy and paste relevant sections from the manuscript (include quotes in quotation marks "like this" to indicate direct quotes from your manuscript), or elaborate on this item by providing additional information not in the ms, or briefly explain why the item is not applicable/relevant for your study

Yes. This is Table 3 in the paper.

16) For each group, number of participants (denominator) included in each analysis and whether the analysis was by original assigned groups

16-i) Report multiple “denominators” and provide definitions

Report multiple “denominators” and provide definitions: Report N’s (and effect sizes) “across a range of study participation [and use] thresholds” [1], e.g., N exposed, N consented, N used more than x times, N used more than y weeks, N participants “used” the intervention/comparator at specific pre-defined time points of interest (in absolute and relative numbers per group). Always clearly define “use” of the intervention.

	1	2	3	4	5	
subitem not at all important	○	○	○	○	○	essential

Does your paper address subitem 16-i? *

Copy and paste relevant sections from the manuscript (include quotes in quotation marks "like this" to indicate direct quotes from your manuscript), or elaborate on this item by providing additional information not in the ms, or briefly explain why the item is not applicable/relevant for your study

No, as the tool was a one-off.

16-ii) Primary analysis should be intent-to-treat

Primary analysis should be intent-to-treat, secondary analyses could include comparing only “users”, with the appropriate caveats that this is no longer a randomized sample (see 18-i).

	1	2	3	4	5	
subitem not at all important	○	○	○	○	○	essential

Does your paper address subitem 16-ii?

Copy and paste relevant sections from the manuscript (include quotes in quotation marks "like this" to indicate direct quotes from your manuscript), or elaborate on this item by providing additional information not in the ms, or briefly explain why the item is not applicable/relevant for your study

Your answer

17a) For each primary and secondary outcome, results for each group, and the estimated effect size and its precision (such as 95% confidence interval)

Does your paper address CONSORT subitem 17a? *

Copy and paste relevant sections from the manuscript (include quotes in quotation marks "like this" to indicate direct quotes from your manuscript), or elaborate on this item by providing additional information not in the ms, or briefly explain why the item is not applicable/relevant for your study

Yes. "Postintervention Differences Among DA Groups
Immediately after the intervention, there were no differences among the 3 DA groups for the primary outcome of lifestyle intentions or secondary outcomes of risk perception, credibility, emotional response, or decisional conflict. For hypothesis 1, the combined DA groups did not differ from the control group for any outcome (Table 5). For hypothesis 2, there was no difference between standard and literacy-sensitive DAs for any outcome (Table 6). There were significant interactions between DA and health literacy for intention to talk to a doctor about medication (P=.02) and emotional responses (positive P=.01; negative P=.006). Participants with lower health literacy who received literacy-sensitive DA had a more negative or less positive emotional response and had stronger intentions to see a doctor about medication compared with the other groups (Table 6).
4-Week Differences Among DA Groups
At follow-up after 4 weeks, there were no significant differences between the control and DA groups for most self-reported behaviors. However, the literacy-sensitive DA group had higher fruit consumption compared with both the control (difference in predicted counts=0.69, 95% CI 0.32-1.06; P<.001) and standard DA groups (difference in predicted counts=0.48, 95% CI 0.11-0.86]; P=.01). The DA groups were more likely to know whether their risk was low, medium, or high than the control group (literacy-sensitive DA: incident rate ratio [IRR]=1.28, 95% CI 1.04-1.58; P=.02 and standard DA: IRR=1.41, 95% CI 1.14-1.74; P=.002). The standard DA group was more likely to know their exact risk percentage result compared with the control group (IRR=3.25, 95% CI 1.31-8.07; P=.01; Table 5). There were significant differences among DA groups by health literacy levels for self-reported calls to the Heart Foundation helpline (P<.001) and verbatim knowledge of CVD percentage risk at follow-up (P<.001). None of the participants with low health literacy reported calling the helpline or remembered their exact CVD risk in the control group. Standard DA increased both outcomes in all health literacy groups, and literacy-sensitive DA increased both outcomes in the low and high health literacy groups but not in the medium group (Table 6).
Postintervention Differences Among Heart Age Groups
Immediately after the intervention, there were no differences between the 2 heart age groups in the primary outcome of lifestyle intentions or secondary outcomes of risk perception or decisional conflict. For hypothesis 3, the heart age group was less likely to have a positive emotional response (mean difference −0.56, 95% CI −0.88 to −0.24; P=.001; Cohen d=0.23), less likely to perceive the message as credible (mean difference −0.20, 95% CI −0.35 to −0.05; P=.01; Cohen d=0.17), and more likely to know whether their risk was low, medium, or high (odds ratio 2.03, 95% CI 1.33-3.08; P=.001), compared with the percentage risk only group (Table 7). When the heart age result was older, there were significant differences indicating less positive (mean difference −0.75, 95% CI −1.19 to −0.31; P=.001; Cohen d=0.31) and more negative (mean difference 0.57, 95% CI 0.12 to 1.02; P=.01; Cohen d=0.23) emotional responses, lower credibility (mean difference −0.29, 95% CI −0.49 to −0.09; P=.005; Cohen d=0.25) and higher perceived risk level (odds ratio 2.11, 95% CI 1.31-3.39; P=.002) when heart age was shown. No such differences were found in those who received the same age or younger results (Table 7).
4-Week Differences Among Heart Age Groups
At the 4-week follow-up, there were no significant differences among the heart age groups in terms of lifestyle behavior change, seeing a doctor for a heart health check, or gist knowledge of risk level (Table 7). Unsurprisingly, heart age led to greater gist knowledge of

heart age (IRR 2.90, 95% CI 2.10-3.99; P<.001) and verbatim knowledge of heart age (IRR 18.13, 95% CI 4.36-75.48; P<.001) compared with those who were not shown their heart age, but there was no difference between the heart age and percentage risk only groups for knowledge of percentage risk. Within the heart age group that saw both risk formats, participants were more likely to have verbatim knowledge of their heart age (11%) than their percentage risk (6%, chi-square test for paired proportions by McNemar: $\chi 2$ =6.1; P=.01, difference in proportions 5.4%, 95% CI 0.8%-10.0%). When the heart age result was older, there were significant differences indicating more vigorous exercise (mean difference 0.58, 95% CI 0.09-1.07; P=.02), more vegetable serves (mean difference 0.57, 95% CI 0.05-1.09; P=.032), higher chance of meeting guidelines for exercise (IRR 1.23, 95% CI 1.05-1.45; P=.01) and diet (IRR 1.48, 95% CI 1.00-2.18; P=.048), when heart age was shown. When the heart age result was the same or younger than their current age, there were significant differences, indicating fewer soft drink serves (mean difference −0.34, 95% CI −0.61 to −0.07; P=.012) and a higher chance of calling the Heart Foundation helpline (IRR 12.66, 95% CI 1.76 to 4.03; P<.001), when heart age was shown (Table 7).

17a-i) Presentation of process outcomes such as metrics of use and intensity of use

In addition to primary/secondary (clinical) outcomes, the presentation of process outcomes such as metrics of use and intensity of use (dose, exposure) and their operational definitions is critical. This does not only refer to metrics of attrition (13-b) (often a binary variable), but also to more continuous exposure metrics such as “average session length”. These must be accompanied by a technical description how a metric like a “session” is defined (e.g., timeout after idle time) [1] (report under item 6a).

	1	2	3	4	5	
subitem not at all important	○	○	○	○	○	essential

Does your paper address subitem 17a-i?

Copy and paste relevant sections from the manuscript (include quotes in quotation marks "like this" to indicate direct quotes from your manuscript), or elaborate on this item by providing additional information not in the ms, or briefly explain why the item is not applicable/relevant for your study

Your answer

17b) For binary outcomes, presentation of both absolute and relative effect sizes is recommended

Does your paper address CONSORT subitem 17b? *

Copy and paste relevant sections from the manuscript (include quotes in quotation marks "like this" to indicate direct quotes from your manuscript), or elaborate on this item by providing additional information not in the ms, or briefly explain why the item is not applicable/relevant for your study

No, this is not relevant for this paper.

18) Results of any other analyses performed, including subgroup analyses and adjusted analyses, distinguishing pre-specified from exploratory

Does your paper address CONSORT subitem 18? *

Copy and paste relevant sections from the manuscript (include quotes in quotation marks "like this" to indicate direct quotes from your manuscript), or elaborate on this item by providing additional information not in the ms, or briefly explain why the item is not applicable/relevant for your study

Yes. See above for more detail.

18-i) Subgroup analysis of comparing only users

A subgroup analysis of comparing only users is not uncommon in ehealth trials, but if done, it must be stressed that this is a self-selected sample and no longer an unbiased sample from a randomized trial (see 16-iii).

	1	2	3	4	5	
subitem not at all important	○	○	○	○	○	essential

Does your paper address subitem 18-i?

Copy and paste relevant sections from the manuscript (include quotes in quotation marks "like this" to indicate direct quotes from your manuscript), or elaborate on this item by providing additional information not in the ms, or briefly explain why the item is not applicable/relevant for your study

Your answer

19) All important harms or unintended effects in each group

(for specific guidance see CONSORT for harms)

Does your paper address CONSORT subitem 19? *

Copy and paste relevant sections from the manuscript (include quotes in quotation marks "like this" to indicate direct quotes from your manuscript), or elaborate on this item by providing additional information not in the ms, or briefly explain why the item is not applicable/relevant for your study

No, no harms were found in this study.

19-i) Include privacy breaches, technical problems

Include privacy breaches, technical problems. This does not only include physical “harm” to participants, but also incidents such as perceived or real privacy breaches [1], technical problems, and other unexpected/unintended incidents. “Unintended effects” also includes unintended positive effects [2].

	1	2	3	4	5	
subitem not at all important	○	○	○	○	○	essential

Does your paper address subitem 19-i?

Copy and paste relevant sections from the manuscript (include quotes in quotation marks "like this" to indicate direct quotes from your manuscript), or elaborate on this item by providing additional information not in the ms, or briefly explain why the item is not applicable/relevant for your study

Your answer

19-ii) Include qualitative feedback from participants or observations from staff/researchers

Include qualitative feedback from participants or observations from staff/researchers, if available, on strengths and shortcomings of the application, especially if they point to unintended/unexpected effects or uses. This includes (if available) reasons for why people did or did not use the application as intended by the developers.

	1	2	3	4	5	
subitem not at all important	○	○	○	○	○	essential

Does your paper address subitem 19-ii?

Copy and paste relevant sections from the manuscript (include quotes in quotation marks "like this" to indicate direct quotes from your manuscript), or elaborate on this item by providing additional information not in the ms, or briefly explain why the item is not applicable/relevant for your study

Your answer

DISCUSSION

22) Interpretation consistent with results, balancing benefits and harms, and considering other relevant evidence

NPT: In addition, take into account the choice of the comparator, lack of or partial blinding, and unequal expertise of care providers or centers in each group

22-i) Restate study questions and summarize the answers suggested by the data, starting with primary outcomes and process outcomes (use)

Restate study questions and summarize the answers suggested by the data, starting with primary outcomes and process outcomes (use).

	1	2	3	4	5	
subitem not at all important	○	○	○	○	○	essential

Does your paper address subitem 22-i? *

Copy and paste relevant sections from the manuscript (include quotes in quotation marks "like this" to indicate direct quotes from your manuscript), or elaborate on this item by providing additional information not in the ms, or briefly explain why the item is not applicable/relevant for your study

Yes. "Principal Findings
We used both a mixed method development and evaluation process to produce a CVD DA that is effective for improving verbatim and gist knowledge of CVD risk and fruit consumption after 4 weeks. The resulting intervention is a scalable eHealth tool suitable for people with varying levels of health literacy. This consumer tool will supplement a GP version for use within consultations, providing GPs with a clear action for their patients to follow up when lifestyle change is recommended. This paper provides an example of how to apply literacy-sensitive design principles to evidence-based decision-making and behavior change tools. The results show that literacy-sensitive DAs can support people with lower health literacy in making informed decisions, while still being suitable for the general population.
Comparison With Previous Work
A recent review of DAs for people with lower health literacy showed that DAs that use health literacy design strategies lead to improved knowledge, decisional conflict, and decision-making outcomes. Furthermore, DAs that used explicit strategies to reduce cognitive burden showed greater improvements in knowledge for people with low health literacy and from disadvantaged backgrounds. The review highlighted the need for more consideration of health literacy in DA development. This study addresses these findings in the context of CVD prevention for the first time.
We observed several interactions with health literacy, showing the importance of considering this as a covariate when investigating shared decision-making and behavior change outcomes. The literacy-sensitive version of the DA produced more negative emotional responses and greater intention to speak to a doctor about medication options to reduce CVD risk among those with lower health literacy. This may reflect risk and choice awareness in this group if they had not previously considered themselves to have risk factors for heart disease that could be addressed with preventive medication. As this sample was predominantly low-risk, we would not want a DA to lead to greater actual medication uptake in this group; however, speaking with a physician about risk and how to reduce it may be a positive outcome in line with guidelines to assess risk in this age group.
We replicated previous DA studies by finding increased knowledge of risk among the DA groups compared with the control group. We also replicated our previous finding that a literacy-sensitive action plan can improve diet outcomes across different levels of health literacy, although this was more marked for people with low health literacy.
This study also replicated several heart age effects found in reviews of previous research, in that it leads to a more negative emotional response, increased gist and verbatim knowledge of heart age, but not percentage risk, and reduced credibility, but is neutral for lifestyle change overall. Our subgroup analyses suggest that more nuanced study designs are required to better understand the effects of heart age. First, among those who were shown their heart age, gist knowledge of percentage risk initially improved, but after 4 weeks, gist and verbatim knowledge were higher for heart age than for percentage risk. Previous studies have shown that people with an older heart age may react defensively and focus on other information, such as a low short-term risk level, which in turn may reduce their credibility of the risk result. Analyses of people who received an older heart age result suggest that it may be useful as a marketing tool to gain attention and initiate behavior change, but knowledge of heart age did not translate to knowledge of risk. For the intended purpose of a DA to be used in a clinical context, the focus must be on validated risk results to make informed decisions about medication. Therefore, we decided to use the non–heart age version of the literacy-sensitive DA in future research in general practice. However, web-based heart age tools can incorporate DA and action plan elements with no detrimental effects."

22-ii) Highlight unanswered new questions, suggest future research

Highlight unanswered new questions, suggest future research.

	1	2	3	4	5	
subitem not at all important	○	○	○	○	○	essential

Does your paper address subitem 22-ii?

Copy and paste relevant sections from the manuscript (include quotes in quotation marks "like this" to indicate direct quotes from your manuscript), or elaborate on this item by providing additional information not in the ms, or briefly explain why the item is not applicable/relevant for your study

Yes. "Future trials need to be designed to isolate older heart age results and follow-up behavior over time. In considering how to power such trials, researchers will need to consider how the specific heart age tool they use is calibrated for the intended population (eg, approximately 50% older in our sample using the New Zealand method vs approximately 80% in the Australian/United Kingdom Heart Foundation tool). The primary outcomes also need to be considered carefully. Most heart age research has been conducted with a primary outcome of immediate lifestyle change intentions, where we found no differences. More research could be done to verify the self-reported behavior change among people receiving older heart age results we observed after 4 weeks, using more objective measures such as pedometers.

The end-user interviews were helpful for improving simple navigation and wording issues in the literacy-sensitive version of the DA, but there were some larger issues that could not be resolved using a web-based tool. Most users did not know their blood pressure or cholesterol results; however, even if they had been assessed recently, they had difficulty understanding where different numbers should be entered. This was particularly difficult for cholesterol results in pathology test reports. Therefore, we will test the final revised tool in clinical practice to address the issue of unknown blood pressure and cholesterol, which reduces the accuracy and limits the display of options in line with the current medication guidelines. This tool will be integrated with additional Heart Foundation resources to improve other lifestyle outcomes."

20) Trial limitations, addressing sources of potential bias, imprecision, and, if relevant, multiplicity of analyses

20-i) Typical limitations in ehealth trials

Typical limitations in ehealth trials: Participants in ehealth trials are rarely blinded. Ehealth trials often look at a multiplicity of outcomes, increasing risk for a Type I error. Discuss biases due to non-use of the intervention/usability issues, biases through informed consent procedures, unexpected events.

	1	2	3	4	5	
subitem not at all important	○	○	○	○	○	essential

Does your paper address subitem 20-i? *

Copy and paste relevant sections from the manuscript (include quotes in quotation marks "like this" to indicate direct quotes from your manuscript), or elaborate on this item by providing additional information not in the ms, or briefly explain why the item is not applicable/relevant for your study

Yes. "A limitation is that the web-based panel sample may not be representative of the general population and may better reflect users of web-based heart age tools than patients presenting to primary care for CVD risk assessment. Furthermore, many participants did not know their blood pressure and cholesterol levels, which may have affected their response to the DA because of a less accurate CVD risk result. However, the use of averages reflects the approach used in currently available consumer tools for CVD risk assessment. Different countries also use different CVD risk models or heart age algorithms, which may affect the results given the differences we observed in the older heart age sample. We conducted a large number of analyses on multiple outcomes; however, given the exploratory nature of the study, we did not make adjustments for multiple comparisons. The study was powered by moderate effect sizes and therefore may have lacked the power to detect more subtle differences; however, these findings will be useful for informing sample size calculations for future studies. Finally, we used validated outcomes where possible but behavior changes were self-reported. Future research on heart age should use objective measures over time"

21) Generalisability (external validity, applicability) of the trial findings

NPT: External validity of the trial findings according to the intervention, comparators, patients, and care providers or centers involved in the trial

21-i) Generalizability to other populations

Generalizability to other populations: In particular, discuss generalizability to a general Internet population, outside of a RCT setting, and general patient population, including applicability of the study results for other organizations

	1	2	3	4	5	
subitem not at all important	○	○	○	○	○	essential

Does your paper address subitem 21-i?

Copy and paste relevant sections from the manuscript (include quotes in quotation marks "like this" to indicate direct quotes from your manuscript), or elaborate on this item by providing additional information not in the ms, or briefly explain why the item is not applicable/relevant for your study

Your answer

21-ii) Discuss if there were elements in the RCT that would be different in a routine application setting

Discuss if there were elements in the RCT that would be different in a routine application setting (e.g., prompts/reminders, more human involvement, training sessions or other co-interventions) and what impact the omission of these elements could have on use, adoption, or outcomes if the intervention is applied outside of a RCT setting.

	1	2	3	4	5	
subitem not at all important	○	○	○	○	○	essential

Does your paper address subitem 21-ii?

Copy and paste relevant sections from the manuscript (include quotes in quotation marks "like this" to indicate direct quotes from your manuscript), or elaborate on this item by providing additional information not in the ms, or briefly explain why the item is not applicable/relevant for your study

Your answer

OTHER INFORMATION

23) Registration number and name of trial registry

Does your paper address CONSORT subitem 23? *

Copy and paste relevant sections from the manuscript (include quotes in quotation marks "like this" to indicate direct quotes from your manuscript), or elaborate on this item by providing additional information not in the ms, or briefly explain why the item is not applicable/relevant for your study

Yes. "Australian New Zealand Clinical Trials Registry ACTRN12620000806965"

24) Where the full trial protocol can be accessed, if available

Does your paper address CONSORT subitem 24? *

Cite a Multimedia Appendix, other reference, or copy and paste relevant sections from the manuscript (include quotes in quotation marks "like this" to indicate direct quotes from your manuscript), or elaborate on this item by providing additional information not in the ms, or briefly explain why the item is not applicable/relevant for your study

Yes. "Australian New Zealand Clinical Trials Registry ACTRN12620000806965"

25) Sources of funding and other support (such as supply of drugs), role of funders

Does your paper address CONSORT subitem 25? *

Copy and paste relevant sections from the manuscript (include quotes in quotation marks "like this" to indicate direct quotes from your manuscript), or elaborate on this item by providing additional information not in the ms, or briefly explain why the item is not applicable/relevant for your study

Yes. "This study was funded by a Vanguard grant from the National Heart Foundation of Australia (ID 102215)."

X27) Conflicts of Interest (not a CONSORT item)

X27-i) State the relation of the study team towards the system being evaluated

In addition to the usual declaration of interests (financial or otherwise), also state the relation of the study team towards the system being evaluated, i.e., state if the authors/evaluators are distinct from or identical with the developers/sponsors of the intervention.

	1	2	3	4	5	
subitem not at all important	○	○	○	○	○	essential

Does your paper address subitem X27-i?

Copy and paste relevant sections from the manuscript (include quotes in quotation marks "like this" to indicate direct quotes from your manuscript), or elaborate on this item by providing additional information not in the ms, or briefly explain why the item is not applicable/relevant for your study

Yes. "C Bonner advises the Heart Foundation on health literacy and risk communication issues"

About the CONSORT EHEALTH checklist

As a result of using this checklist, did you make changes in your manuscript? *

- ○ yes, major changes
- ○ yes, minor changes
- ◉ no

What were the most important changes you made as a result of using this checklist?

Your answer

How much time did you spend on going through the checklist INCLUDING making changes in your manuscript *

I spent about an hour and a half going through this checklist. It is too late to make changes to the manuscript as I was only made aware of this checklist at the final proofreading stage.

As a result of using this checklist, do you think your manuscript has improved? *

- ○ yes
- ◉ no
- ○ Other:

Would you like to become involved in the CONSORT EHEALTH group?

This would involve for example becoming involved in participating in a workshop and writing an "Explanation and Elaboration" document

- ○ yes
- ○ no
- ○ Other:

Any other comments or questions on CONSORT EHEALTH

Your answer

STOP - Save this form as PDF before you click submit

To generate a record that you filled in this form, we recommend to generate a PDF of this page (on a Mac, simply select "print" and then select "print as PDF") before you submit it.

When you submit your (revised) paper to JMIR, please upload the PDF as supplementary file.

Don't worry if some text in the textboxes is cut off, as we still have the complete information in our database. Thank you!

Final step: Click submit !

Click submit so we have your answers in our database!

Submit

Clear form

Never submit passwords through Google Forms.

This content is neither created nor endorsed by Google. Report Abuse - Terms of Service - Privacy Policy

Google Forms
